# Springback effect of ambient-pressure-dried silica aerogels: nanoscopic effects of silylation revealed by *in situ* synchrotron X-ray scattering†

**DOI:** 10.1039/d3na00584d

**Published:** 2023-11-28

**Authors:** Fabian Zemke, Ernesto Scoppola, Ulla Simon, Maged F. Bekheet, Wolfgang Wagermaier, Aleksander Gurlo

**Affiliations:** a Technische Universität Berlin, Faculty III Process Sciences, Institute of Materials Science and Technology, Chair of Advanced Ceramic Materials Straße des 17. Juni 135 10623 Berlin Germany https://www.tu.berlin/ceramics fabian.zemke@ceramics.tu-berlin.de +49 30 314 22653; b Department of Biomaterials, Max Planck Institute of Colloids and Interfaces Am Mühlenberg 1 14476 Potsdam Germany https://www.mpikg.mpg.de/biomaterials ernesto.scoppola@mpikg.mpg.de +49 331 567 9259

## Abstract

Ambient pressure drying (APD) allows for synthesizing aerogels without expensive and sophisticated equipment for achieving supercritical conditions. Since APD does not eliminate the capillary stress that is induced by the liquid/vapour phase boundary, the shrinkage during drying needs to be prevented or reversed. The re-expansion of the silylated silica gels during drying is commonly referred to as the springback effect (SBE). The SBE is not only important for producing aerogels *via* APD, but is also a fascinating phenomenon, since it is accompanied by a significant volume change unusual for rigid ceramics. Synchrotron X-ray scattering has proven to be especially effective for the investigation of the volume change of these fractal silica structures on different length scales. In this work, we follow the drying, shrinkage, and (partial) re-expansion of various monolithic samples *in situ* to explore the occurrence of the SBE. For this purpose, various silylation agents, *i.e.*, hexamethyldisilazane, trimethylchlorosilane, and triethylchlorosilane were used to investigate different shrinkage and re-expansion behavior. A scattering model was used to extract additional information of the evolving primary particle size, correlation length, fractal dimension, and other intensity contributions of the silica network and the hexane. While the primary particles pointed towards a relaxation at near molecular size, they were likely not involved in the SBE. However, structures near the size of the correlation length could be essential for the occurrence of this phenomenon. These findings may lead to the origin of this interesting phenomenon, as well as a better understanding of the production of APD aerogels.

## Abstract 2

During ambient pressure drying of silica aerogels, shrinkage and re-expansion is observed, commonly known as the springback effect. *In situ* synchrotron X-ray scattering gives insights into the nanostructural evolution of drying gels.

## Graphical abstract

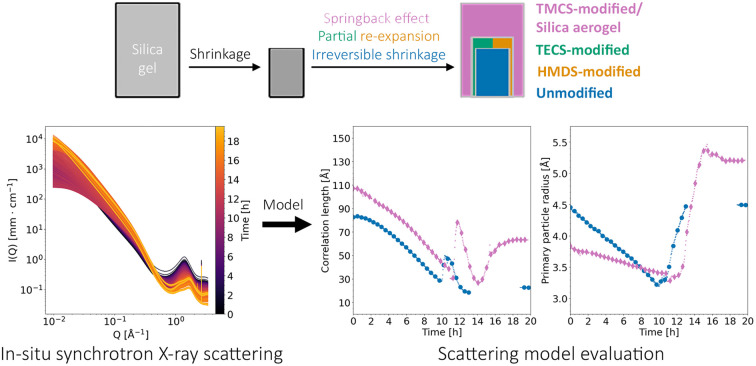

## 1. Introduction

Aerogels are nanostructured materials with exceptional properties, *e.g.*, very low bulk densities, high porosities, specific surface areas, and low thermal conductivities.^1^ While metal,^2^ organic,^3,4^ ceramic or composite aerogels are known;^5–8^ silica aerogels remain the most studied system, making it worthwhile to explore the fundamentals of the aerogel formation and structure.^6,9–11^ Furthermore, the preceding sol–gel process allows for tailoring many of the properties of this inorganic porous network, such as the bulk density, and the size of the elementary particles.^12^

Since a material may only be called an aerogel if the gel network experiences minor or no volume changes during drying,^13^ specific precautions must be taken to minimize the shrinkage of the material. For silica aerogels, this can be achieved by either supercritical drying (SCD) or ambient pressure drying (APD).^14^ Shrinkage of the material is caused by capillary pressure in the pores when a liquid/vapour phase boundary is present.^15^ As described by Laplace–Young's equation, the capillary pressure is increasing with a higher surface tension of the liquid, and lower network's pore sizes, as well as being affected by the contact-angle of the liquid with the gel network.^16^ SCD eliminates this phase boundary, attaining supercritical conditions for the involved fluids.^15^ On the other hand, APD relies on either preventing or minimizing the shrinkage by strengthening the structure, adjusting the network geometry, slowing down the drying time significantly or making the shrinkage reversible.^15,17,18^ The latter can be achieved by surface modification of the gel with silylation agents such as trimethylchlorosilane (TMCS).^19^ This re-expansion introduced by silylation is generally referred to as the springback effect (SBE).^20^ Depending on the used precursors, monolithic specimens may also be achieved by other surface modification agents, *e.g.*, hexamethyldisilazane (HMDS).^19^

The evaporation of the liquid from the gel network is the main driving factor for the shrinkage and re-expansion of the material. The drying of a gel network is discussed intensively by Brinker and Scherer, who categorized the process into distinct steps: (I) a constant rate period where the volume of shrinkage is equal to the evaporated solvent; (II) the critical point, where the network stiffens and shrinkage stops; (III) a first falling rate period, where the solvent retracts into the network, leaving a layer of liquid; (IV) and a second falling rate period dominated by evaporation limited by diffusion.^21^ Although the evaporation of different solvents (*e.g.*, water, ethanol, acetone, hexane, benzene) from porous silica was explored in the past,^18,22^ it remains challenging to correlate the macroscopic shrinkage of the material to nanoscopic changes. The nanoscopic structure may be described by structural parameters on different length scales, *i.e.*, the fractal dimension, the size of clusters and primary particles, which can be evaluated by small-angle and wide-angle X-ray scattering (SAXS/WAXS).^23^ These structural features give rise to a specific scattering profile.

The fractal dimension is a term typically used in mathematics, where Mandelbrot describes a fractal as an object with self-similarity, where each fractional object is geometrically alike the entire object,^24^ having no scale limitations. Besides these theoretical considerations, objects in nature are frequently called fractal even though the range is finite.^25^ Silica aerogels are often termed fractal structures,^26^ where the network has a self-similarity over an order of magnitude.^27^ In this instance, it describes a mass-related self-similarity, also referred to as mass fractal, which may be interpreted as a density of the silica structure.^26^ Similarly, the surface roughness can be described by a surface fractal in the Porod region.^28,29^ Furthermore when correlated with computer models, the fractal dimension may give insights on the initial aggregation behaviour of the silica network,^26,30^ as well as influences of synthesis parameters such as the pH value.^31^

This fractality is limited by the skeletal and the bulk density of the silica network, as described in the literature.^12^ Furthermore, the silica network can be described by the size of clusters and primary particles. While the primary particles are considered to be the elementary structural units that form during the initial gel formation,^14,32^ clusters are often called secondary particles and aggregate from the primary particles.^33^ These two parameters can be evaluated from the crossover of the small-angle scattering region to the fractal dimension, and from the fractal dimension to the Porod region.^34^

Silica aerogels and the distinct synthesis steps have been investigated by SAXS and WAXS in the past. To this end, the sol to gel transformation was severely influenced by the pH values during the synthesis, *i.e.*, the acid and base catalysis.^35^ In another work, the impact of synthesis parameters such as the temperature and pH on the gelling of mesoporous silica with micelles was followed *in situ*, showing a swelling of the micelles and different growth of the network.^36^ Additionally, the gelling was investigated with *in situ* SAXS, starting with an already formed sol of distinct morphology, showing influences of the pH value, the concentration and size of the colloidal particles on the aggregation behavior.^37^ Additionally, the use of X-ray scattering, modelling and machine learning was shown to be promising in the literature, but would require larger datasets for the usage.^23^ While fiber-reinforcement or SCD of the material may improve the production of monolithic samples by limiting its shrinkage,^38,39^ it prevents the full investigation of the SBE. On the contrary, it was shown that it might be possible to follow the SBE by studying samples produced by SCD, as the strain recovery resembled this effect.^40^ Nonetheless, this approach skips over the evolution of the structure during drying and bypasses the stresses inside the material.

In our previous work on unmodified and TMCS-modified silica gels we have shown that the SBE correlates with structural features determined by *in situ* synchrotron X-ray scattering. In this regard, it was shown that a recovery of the fractal slope values was only visible for samples that recovered their original geometry. Furthermore, the Porod slope evolution was attributed to the SBE, indicating a change from fractally rough to a sharp interface.^41^ Hereafter, we compared the effect of the commonly used silylation agent TMCS to HMDS, which might be an economical substitute. Moreover, triethylchlorosilane (TECS) was chosen for comparison, as the bigger molecular size was expected to influence the SBE. While TMCS showed the SBE, the other silylation agents lead to only partial re-expansion. The qualitative content of surface silylation was determined, showing the successful modification for the TMCS-modified gel, and some remaining silanol end groups for the HMDS-modified and TECS-modified gels. Moreover, these dried samples were investigated *ex situ* by X-ray scattering and further evaluated using a fractal scattering model, which showed a higher primary particle size for the TMCS-modified in comparison to the HMDS and TECS gels.^42^

This study combines and expands on the insights from the previous works by investigating gels without surface modification and with various silylation agents (*i.e.*, HMDS, TECS, TMCS) *in situ* during drying by means of synchrotron X-ray scattering coupled with digital imaging. This facilitated comparing gels that showed no volume recovery, partial re-expansion, or the full SBE, for the unmodified, HMDS-modified and TECS-modified, as well as the TMCS-modified gels, respectively. The *in situ* scattering data was analysed further by creating a scattering model assuming intensity contributions of the silica backbone, the drying solvent inside the network, as well as a contribution of polydisperse spherical primary particles in a fractal constellation. This allowed tracking changes of the size of primary particles and correlation length, the fractal dimension, as well as the volume percentage of solvent inside the pore network during the shrinkage and re-expansion. Moreover, since samples with varying degrees of re-expansion were investigated, effects of the silylation can be distinguished from direct influences of the SBE. Ultimately, this allows providing structural insights during the SBE, which could improve the APD synthesis of monolithic silica aerogels.

## 2. Results and discussion

### 2.1. *In situ* X-ray scattering with coupled macroscopic changes

**Fig. 1:**
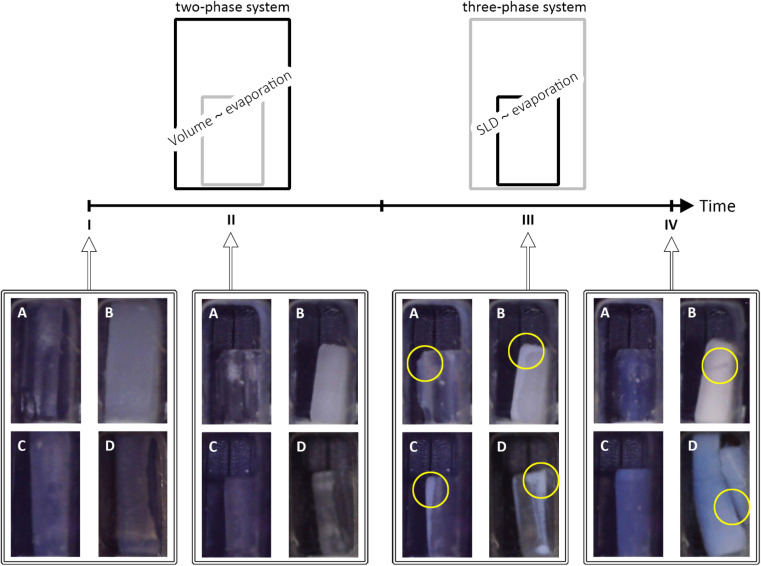
Schematic of the four main sample states (I–IV) over time with selected photographs of the unmodified UN (A), hexamethyldisilazane-modified HM (B), triethylchlorosilane-modified TE (C), and trimethylchlorosilane-modified TM (D) samples are shown. The samples which were completely filled by hexane (I) shrunk proportionally to the evaporation of the solvent (II). Afterwards, the photographs showed a loss in transparency at which point air was entering the system and the scattering length density (SLD) of the solvent was related to the evaporation of the solvent (III), finally ending with a dried sample (IV). Therefore, there was a change from a two-phase system (silica-hexane, I/II) to a three-phase system (silica-hexane-air, III), and finally a two-phase system (silica-air, IV). Likewise, The photographs (width of the individual insets 7 mm ± 0.3 mm) were extracted from the serial photographs throughout the experiment. Yellow circles in the photographs highlight the loss in transparency (III), and crack formation (IV) in the gels.

The structural features of unmodified (UN), and surface-modified samples with hexamethyldisilazane (HM), triethylchlorosilane (TE), and trimethylchlorosilane (TM) were investigated by means of *in situ* SAXS/WAXS to show changes of the gels during the shrinkage and re-expansion. For this purpose, cuboid-shaped gel samples were synthesized as reported previously,^42^ and stored in hexane. Prior to the measurements, the specimens were placed in a measurement cell, which was covered by a lid to keep premature evaporation of the hexane to a minimum. Directly before the measurements, this lid was exchanged for a valve with a defined opening. During the synchrotron measurements, photographs of the samples were taken to bring together macroscopical changes with the scattering profiles. As visualized in Fig. 1, the drying of the samples can be divided into four states, denoted from I to IV. Here, I, and IV refer to the first and the last measurement, respectively, whereas II and III are ranges of the measurement series. The first range (II) refers to the shrinkage of a drying specimen, which was filled completely by hexane, as indicated by a high order of transparency. It was assumed that the loss of volume of the gel was directly proportional to the loss of hexane, resulting in a two-phase system of silica and hexane at every point in time. This phenomenon is discussed extensively by Brinker and Scherer and is often referred to as the constant rate period.^21^ According to the literature, the pores will empty in declining size, and the shrinkage is proportional to the size and volume of mesopores.^18^ Digital images of the samples in the second range (III) showed a change in transparency, where mostly the edges became translucent pointing towards air entering the system as reported in the literature,^43^ which resulted in a three-phase system of silica, hexane, and air. As shown in the literature, the structure stiffens and endures the capillary pressures, resulting in a maximum shrinkage. The constant rate period is replaced by the first falling rate period where the solvent withdraws into the porous structure, leaving a film of liquid on the inner surface of the network.^21^ The scattering length density (SLD) of the solvent changed from hexane to a mixture of hexane and air until the specimen was completely dried. Supposedly, the solvent SLD was proportional to the evaporation of hexane. The macroscopic changes of the samples are also shown in Fig. 1, where all samples showed shrinkage until the cutoff point (II/III). Following, the UN sample was not changing noticeably in size, but changed its color and became blue, and transparent. The HM and TE samples showed a slight re-expansion but differed in color. While the HM sample adopted a white-opaque appearance, the TE sample was behaving similar to the UN sample. Finally, the TM sample showed an almost full re-expansion but concurrent cracking of the material, turning blue-opaque which is typical for aerogels.^44^ The TM sample will therefore be considered as the main example of the SBE, whereas the UN sample is the reference sample, and the HM and TE samples show an intermediate step. The nanoscopic structural changes of these four stages I–IV were resolved by means of SAXS/WAXS.

**Fig. 2:**
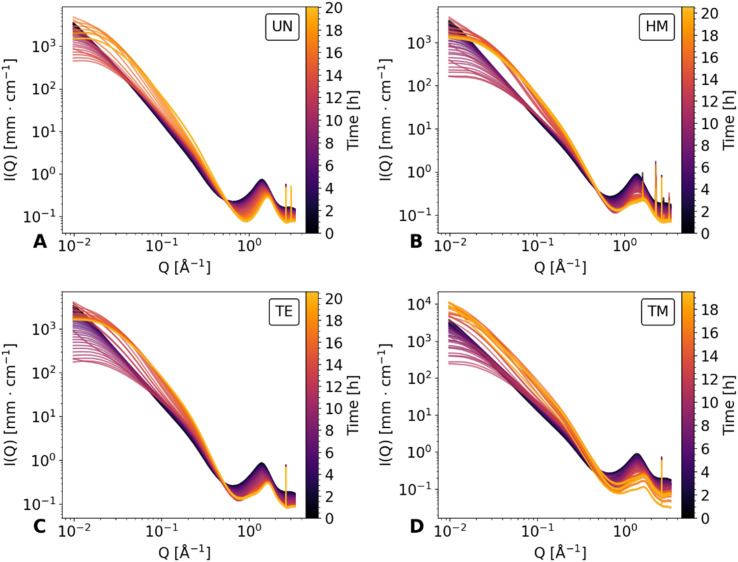
*In situ* SAXS diagrams of the unmodified UN (A), hexamethyldisilazane-modified HM (B), triethylchlorosilane-modified TE (C), and trimethylchlorosilane-modified TM (D) samples over the duration of the drying. For better visualization, only every 20th measurement was plotted. Additionally, the elapsed time is color-coded and shown as a colorbar on the right side of each scattering diagram.

The drying of the cuboid samples was followed by synchrotron X-ray scattering in a *Q* range of 0.007 Å^−1^ to 4 Å^−1^ over the duration of roughly 20 hours. Data are shown for every 20th measurement in Fig. 2 and for the full dataset in Fig. S1.† The measurements were normalized for time, flux of the beam, transmission, and background subtracted accordingly, but could not be adjusted for the change in width of the specimens. In the following, the scattering profile will be explained, noting conspicuous features from WAXS to the SAXS region, and thus in the real space, from small to bigger objects (*ca.* 1.6 Å to 890 Å).

At the start of drying, all samples showed a broad peak at roughly 1.37 Å^−1^, which can be attributed to the scattering of hexane. This was confirmed by a separate measurement of a hexane-filled glass capillary, as shown in Fig. S2,† which was compared with the first and last measurements of the TM sample. During the drying, two additional peaks became visible at *ca.* 1.2 Å^−1^ and 1.6 Å^−1^ for the HM, TE, and TM samples, while the UN sample was only showing the peak at roughly 1.6 Å^−1^. This suggested that the peaks were features of the silica backbone, which were overshadowed by the hexane contribution, as reported in a previous study.^41^ The peak at 1.2 Å^−1^ was mainly visible for the surface-modified samples and negligible for the UN sample, indicating that this is characteristic of the silylation of the material. This indication is further extended by the fact that the intensity of this peak was noticeably different for the HM and TM, which share the same resulting modification, in comparison to the TE samples. Additional sharp peaks became visible in the WAXS region for all samples, which were not reported beforehand. These could be nanograins captured by the beam or could be signs of local crystallization. While further investigation of these crystalline peaks could be worthwhile, they were not the focus of this study.

Directly beside the broad peaks in the WAXS region, in the *Q* region of 0.31 Å^−1^ and 0.45 Å^−1^ (*i.e.*, Porod region) a linear decay can be seen in the double logarithmic plot. Previous works have shown that a proper slope evaluation in the specific *Q* region requires preliminary determination of a *Q*-dependent scattering contribution of the solvent.^23^ Consequently, the contribution of different intensities has to be considered to fully investigate the structural parameters. Nonetheless, we have shown in our previous work, that subtraction of the hexane signal may be used for a more realistic estimation of the Porod slope.^41^ This evaluation was replicated for the current data and is shown for comparability in Note S1, S2 and Fig. S3–S5.†

By moving towards lower *Q* values, the intermediate *Q* region between 0.03 Å^−1^ and 0.26 Å^−1^ is dominated by another linear decay, the fractal slope.^41^ While all samples showed a linear decay of the fractal region at first, the curvature of scattering profiles changes during drying, flattening for *Q*-values below 0.03 Å^−1^. Furthermore, in the UN, HM, and TE samples flattening of the SAXS region persists until the end of the measurement. On the other hand, the TM sample seemingly recovered its original scattering profile in correlation with the SBE and almost full recovery of the gel geometry. At the same time, the SAXS intensity increased consistently during the evaporation of the solvent, since the SLD difference of the silica/fluid is higher for air than for hexane. A similar effect was previously observed for a dried polyimide aerogel in contrast to a specimen filled with solvent.^45^ As an estimation, the fractal dimension can be evaluated from the slope of the double-logarithmic plot,^25^ as is shown in Fig. S6 and discussed in Note S3.†

### 2.2. Scattering model

#### 2.2.1. Constraints and X-ray transmission

SAXS/WAXS measurements can be further explored by assuming a scattering model with a compiled intensity contribution of the silica network and hexane. To this end, a scattering model was created consisting of three Lorentzian peaks representing the (modified) silica backbone and the hexane in the WAXS region, as well as a fractal contribution in the SAXS region. The latter was originally reported by Teixeira *et al.* and considered spherical, interconnected primary particles,^46^ which was shown in the literature to be a reasonable assumption for the modeling of aerogels.^47^ This combined scattering model allowed us to determine structural parameters, such as the position, half-width at half-maximum (HWHM), and scale of the Lorentzian peaks, as well as the scale, correlation length, fractal dimension, and primary particle radius of the fractal intensity contribution. This intensity contribution constrained the scale of the Lorentzian peaks associated with the silica backbone. A detailed explanation of the model can be found in the Materials and methods section of this work.

**Fig. 3:**
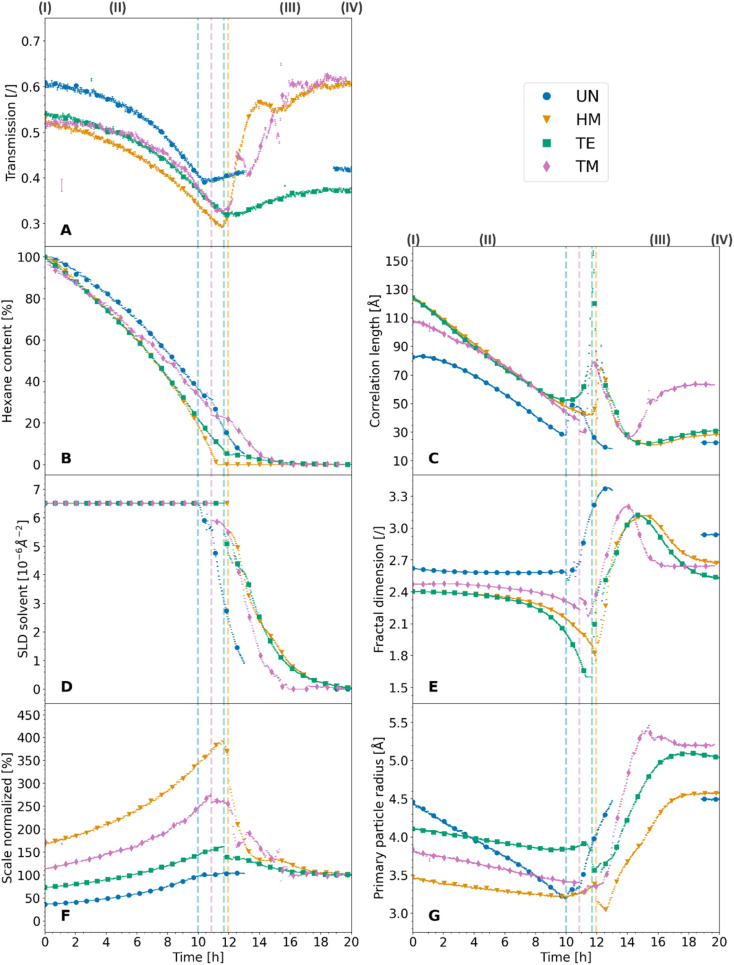
The scattering model data evaluation of the synchrotron X-ray scattering measurements of the unmodified UN (blue, circle), hexamethyldisilazane-modified HM (orange, triangle), triethylchlorosilane-modified TE (green, square), and trimethylchlorosilane-modified TM (magenta, diamond) samples with their corresponding standard deviation (bar line) are shown, as well as the measured transmission of the samples (A), for the duration of the experiment. Additionally, dashed vertical lines in the color of the appropriate samples, show the estimated crossover of the two-phase system (silica-hexane) to a three-phase system (silica-hexane-air). The normalized scale of the hexane peak (B) shows the hexane content assuming a sample filled by hexane, which was completely dried in the end. The scattering length density of the solvent (D) was pure hexane at first, and then was constrained to the hexane peak in the three-phase system. The scale of the fractal contribution (F) was left unrestricted and normalized to 100% at the end of the measurements. The correlation length (C), fractal dimension (E) and the primary particle radius (G) were evaluated from the fractal intensity contributions of the samples.

The X-ray scattering measurements were performed at three different heights for each sample. The calculated transmission, as well as the data evaluation of the scattering model is shown for the middle position in Fig. 3, which shows the same tendencies as the other measurements. Additional model parameters are shown with their results and discussions in Fig. S7 and Note S4.† The other positions are shown for reproducibility in Fig. S8 and S9,† respectively. The goodness of the scattering model is discussed in Note S5 and shown in Fig. S10–S13.† As discussed earlier (Fig. 1), a change from a two-phase to a three-phase system of hexane-silica to hexane-air-silica was presumed within the experiment. This required a split of the batch evaluation for ranges II and III, where the latter introduced an additional constraint for the solvent SLD. The cutoff point, shown as a dashed vertical line in Fig. 3, represents the transition from the two-phase (II) to the three-phase (III) system and it was determined by assessing the digital photographs of the samples as discussed earlier. Interestingly, it correlates with the calculated transmission values over time (Fig. 3A). In fact, all samples followed the same trend, showing a sharp decrease of transmission (II), reaching a minimum value which was similar for the HM, TE, and TM samples, followed by another sample-dependent transmission increase.

While the absorption coefficient values are related to the material, the evolution of the X-ray transmission of the samples under investigation has two main influences. On the one hand, a decrease in the volume of the samples leads to a density increase and a decrease in transmission. On the other hand, the evaporation of the solvent and the exchange of hexane with air decreased the absorption and thereof increased the transmission of the specimen. Since the crossover of the two ranges II and III was at the point of maximum shrinkage, a correlation with the minimum of the transmission values was expected. While the minimum of the transmission was concurrent with the cutoff point for the TE sample, and therefore the macroscopical optical changes, the UN and the TM samples showed this minimum slightly later and the HM sample slightly earlier. Insignificant shifts of the minimum were expected since the macroscopic state of the samples was compared with a small sample volume hit by the synchrotron beam. This could indicate that the probed spot was not yet changing to a three-phase system. The almost full re-expansion and SBE of the TM sample were visible in a recovery of the transmission values. Local variations in the TM transmission data might be attributed to crack formation, causing local and sudden inhomogeneities in sample thickness, which was visible in the photographs (Fig. 1). The sharp increase in transmission (Fig. 3A) for the HM sample was surprising, and it ended at significantly higher values at the end (IV) in comparison to the start (I), which would indicate a significant volume recovery. Since the photographs (Fig. 1) showed only partial re-expansion, this might instead be explained by either a crack or bubble inside the material in the direction of the X-ray beam as can be seen in Fig. 1. These assumptions are coherent with a previous study showing in some instances crack formations for HMDS-modified, TECS-modified, and TMCS-modified samples.^42^ Overall, it was possible to match the transmission values with the sample states.

#### 2.2.2. Normalized hexane content and SLD of the solvent

The normalized hexane content (*i.e.*, the time-dependent hexane content normalized over initial value) throughout the measurements can be seen in Fig. 3B, varying between 100% and 0% for the first (I) and last (IV) measurements, respectively. The evolution of the hexane content can be correlated with the previously explained macroscopic evolution of the samples since evaporation is the main driving factor for capillary pressures and the shrinkage of the material. Consistently with previous studies,^41^ we noticed in particular that after 4 hours (see Fig. S14†) the UN sample exhibited a faster drying velocity than the TM sample, despite that the latter should dry faster because of a bigger surface area. This might be explained by a different affinity of the modified material's surface with the hexane. As shown in the literature, the capillary pressures inside the gel structure are affected by the contact angle of the solvent and the network, especially if a layer of liquid is absorbed on its surface.^15^ It was previously reported that the shrinkage could be further reduced by extending the drying time.^18^ The difference of a few hours of drying time was unlikely to be the main reason for the SBE. Nonetheless, this might be another supporting property. After the cutoff point (II/III), the UN and especially the TM samples demonstrated almost stagnant hexane evaporation. The decrease in the evaporation rate indicates a change in the drying mechanism.^21^ This was followed by a steep decrease in hexane content, where the drying seemed to be accelerated after the point of maximum shrinkage. On the contrary, the HM and the TE samples showed almost 0% hexane content at the cutoff point (II/III), which was implausible. This indicated that these two samples still had a small residual amount of solvent after the measurements since the normalized hexane content was derived from the initial and final hexane content. It was shown in the literature that the drying at the end is achieved by diffusion processes and is heavily influenced by the adsorption of the solvent on the surface of the gel network.^21^ Another explanation could be that the direct constraint of the silica peaks to the volume fraction of the material may lead to an overestimation of the silica contribution to the overall scattering intensity and *vice versa* an underestimation of the hexane contribution.

For consistency, fits were performed by constraining the solvent SLD to the hexane scaling factor after the cutoff point (II/III). Therefore, the SLD of the solvent followed a similar trend of the hexane content evaluation as shown in Fig. 3D. As can be seen in eqn (4), the small-angle intensity is equal to the product between the SLD difference squared, (*ρ*_block_ − *ρ*_solvent_)^2^, the form and structure factors, *P*(*Q*) and *S*(*Q*) respectively, the scale *α*, and the volume fraction *ϕ*.

#### 2.2.3. Normalized scale correlated to volume change

In a previous study, dried samples with comparable properties were investigated, and their porosity was calculated.^42^ The resulting volume fractions were used to set the value of *ϕ* for the dried samples, hence the last measurements (IV), and were kept static throughout the model evaluation. On the other hand, we remind that data were not normalized over the sample width. Therefore, by constraining *ϕ*, we made the decision to set α as a free parameter since the scattered intensity will vary with the sample width and the volume fraction. In Fig. 3F the evolution of small-angle scale normalized over its value for the last data frame is reported. The normalization over the last data frame was chosen because of the ESI† of our previous study, where the skeletal and bulk densities, the chemical environment and surface modification, as well as the microstructure of dried modified gels, comparable to this study, was extensively evaluated.^42^ The normalized scale allowed us to distinguish between shrinkage and re-expansion of the respective samples. Locally sharp variation of the normalized scale might indicate cracking and gaps of the sample, which were also visible in the optical images. Paradigmatically, the TM sample, which shows the SBE, started at roughly 114% (I), rose to *ca.* 276% (II/III) at the cutoff point, which was the point of maximum shrinkage, and ended up at 100% (IV) again. This translated to a shrinkage to roughly half its size with almost full re-expansion of the material. In contrast, the UN sample started at roughly 37% (I) and increased to *ca.* 100% (II/III), with no further variations for the remaining measurement series. The TE sample could be described as an intermediate sample between the UN and the TM samples. With its starting value of 73% (I), it rose to roughly 163% (II). At the cutoff point (II/III), a small jump in the data set was visible, afterwards decreasing again (IV). The difference between the last and first measurement points shows the irreversible shrinkage of the sample. Surprising was the large increase in the scale of the HM sample, which started at roughly 165% (I), reached *ca.* 394%, followed by a significant drop around the cutoff point (II/III). Afterwards, it monotonically decreased (III/IV). Exhibiting a larger scale in the start in comparison to the end is only feasible if the sample shows a higher re-expansion than its original volume, which was disproven by the optical photographs. This indicates severe cracking of the material, where the X-ray beam was either hitting a bubble or a crack, grazing the sample only slightly.

The normalized scale seemingly correlated with other parameters of the investigation, such as the transmission values of the samples and the hexane content. In this regard, an increase in the scale entailed a decrease in the transmission and *vice versa*. This was not surprising as both parameters are susceptible to changes in the volume of the samples. However, since the transmission is also related to the hexane content, the normalized scale represents the shrinkage and re-expansion of the material better. In that respect, the normalized scale does not contain the hexane content rather, it is correlated to it until the cutoff point (II/III). Since the evaporation of the solvent was the main driving factor for the shrinkage of the material, the anti-proportional relationship between the hexane content and the normalized scale is apparent. As discussed in Note S4,† the scaling factor of the fractal contribution indicated an overestimation of porosity values of a previous study,^42^ though being ambiguous because the data could not be normalized for the sample width. Further normalization over the sample width could also facilitate determining the quantitative degree of re-expansion during the SBE and the amount of irreversible shrinkage by using the normalized scale.

#### 2.2.4. Correlation length, fractal dimension and primary particle size

So far, we have discussed global parameters such as the SLD, scale, and volume fraction, but small-angle modelling provides insight into the structure coded in the form and structure factor. In particular, the latter provides information about the correlation length *ξ* (see eqn (8)) as a parameter of the fractal intensity contribution eqn (2) and it should be correlated to the changes during the shrinkage and re-expansion process. The evolution of the correlation length is shown in Fig. 3C. While all samples showed a similar development, decreasing at first (I/II), showing a sharp increase at the cutoff point (II/III) followed by a sharp decrease (III), some significant differences were visible. The first difference is represented by the different initial *ξ* values: while the UN sample starts at a value of 83 Å, HM, TE, and TM samples started at a value of *ca.* 124 Å/107 Å. This could be an indication of enhanced aging of the UN sample since the growth of primary particles and reorganization of the network can cause a decrease in correlation length, as was shown in the literature.^48^ However, this could also showcase the attached silyl groups, which increased the size of the cluster. Secondly, directly before the cutoff point, the three modified samples exhibited the same correlation length of around 50 Å in contrast to the value recorded for UN samples of approximately 30 Å, which was likely due to the disparity in starting values. The third difference is observed after the sharp decrease following the cutoff point. In fact, the TM sample is the only one exhibiting a non-negligible increase of *ξ* ending at approximately a value of 63 Å. With correlation lengths of roughly 151 Å and 17.5 Å reported in the literature for fumed silica nanoparticles of 12 nm, and disordered mesoporous silica, respectively, the calculated values here can be located between the two.^49^ Moreover, the final value of *ξ* seems to be correlated to macroscopic evolutions: the larger the SBE, the larger the correlation length is. Here, *ξ* might describe the size of the clusters inside the gel network,^50^ which increase in size with the re-expansion of the material. Since a previous study on similar samples showed only slight discrepancies in the degree of surface modification, while demonstrating severely different macroscopic re-expansion, it was suggested that the silylation might not be the sole reason for the SBE.^42^ Moreover, the influence of mechanical properties on the SBE was demonstrated in other work.^40^ The macroscopic re-expansion and occurrence of the SBE could be influenced by processes near the cluster size. On the same line, macroscopic observations and nanoscale structural organization could also be related to the optical properties of the material. To this end, a high translucence was reported for relatively low cluster sizes.^51^ This was the case for the UN, TE, and HM samples. In particular, the first two samples showed a high amount of transparency in the optical images (Fig. 1) and were possibly indicative of inhomogeneities or defects/cracks inside the HM sample, as was reported in the literature.^51^

However, another interpretation of the evolution of *ξ*, which includes the previously mentioned sharp increase at the cutoff point (II/III), is that this parameter refers to the correlation length over which the system cannot be considered fractal anymore.^52^ In this regard, all samples showed a decrease in *ξ* because of the shrinkage of the material until they reached a point of maximum shrinkage. At this point, the silica structure with the porous network filled with hexane might be considered fully dense, such as the mass-related self-similarity enclosed the whole sample, resulting in a spike of *ξ*. Directly afterwards (III), air enters the system, and *ξ* could represent the fractal structure of the silica network covered by a layer of hexane. To this effect, the sharp decrease in correlation length does not show a shrinkage of the material but rather a decrease in the thickness of the hexane layer. This is substantiated by the fact that the macroscopic images, as well as the normalized scale, already showed either re-expansion or irreversible shrinkage while the correlation length still decreased. Finally, the increase in *ξ* (III/IV) for the TM sample could indicate that the fractal structure was restored, whereas the other samples could be considered fractal structures over a significantly lower range. Therefore, the increase of the clusters was superseded by the decrease of the adsorbed solvent layer. Within this line of thought, the difference between starting (I) and final (IV) value of *ξ* could be interpreted as a contribution of irreversible shrinkage and the adsorbed layer of solvent at the surface of the gel network.

As well as the correlation length, the fractal dimension shown in Fig. 3E is another parameter providing insights into the SBE, shrinkage, and re-expansion. At first (I), the UN, HM, TE, and TM samples started at similar values of 2.62, 2.40, 2.40, and 2.47, respectively. Afterwards, the modified samples HM, TE, and TM samples showed a gradual decrease, ending at roughly 1.76, 1.60, and 2.23 values at the transition point of two-phase to the three-phase system (II/III) with 1.60 the minimum boundary of the applied model. On the contrary, the UN sample stagnated until the cutoff point, where the samples experienced the point of maximum shrinkage. Then all samples increased sharply, reaching values of 3.38 (UN), 3.12 (HM), 3.12 (TE), and 3.21 (TM), followed by a strictly monotonous decrease, which was accelerated for the TM sample in comparison to the HM and TE samples, and not well-defined for the UN sample (III). Finally, the samples ended (IV) at values of 2.93 (UN), 2.68 (HM), 2.51 (TE), and 2.64 (TM). Commonly, the fractal dimension is interpreted as a parameter describing the growth model of the material, giving insights into their initial sol to gel aggregation behavior.^30^ On the contrary, this interpretation cannot be applied to the results presented in this work, as the initial aggregation behavior cannot change throughout the drying. Likewise, the fractal dimension is described in the literature as the relative density of the clusters,^12,25^ where an increase in the fractal dimension would refer to an increase in the overall density or interconnectivity of the aerogel network.^9^ While this might be true for a non-evolving material, the evaluation of this work indicates that these statements cannot be used for the *in situ* interpretation of the density without background information of the investigated system. While the samples shrunk, which should increase their density, the calculated fractal dimension values (Fig. 3E) either decreased or stayed constant.

Some of these trends of the evolution in fractal dimension, *i.e.*, a drastic change of fractal dimension near the cutoff point (II/III) for the modified samples, followed a similar trend discussed for the correlation length. A relation between the two parameters was also reported in the literature, where it was shown that both were similarly influenced by synthesis parameters.^53^ As was reported for other materials, dried and samples filled by solvent can be differentiated using the fractal dimension, showing lower mass fractal values for the wet materials in comparison to the dried.^54^ Following a similar interpretation of the fractal dimension evolution compared to the correlation length, the values might be influenced by the hexane. All samples showed macroscopic shrinkage as demonstrated in the photographs. The fact that the fractal dimension does not change in this initial period would indicate that the fractal structure mostly stayed intact, and the structure shrunk to an identical amount to the loss of pore volume. The latter is supported by the initial drying phenomenology reported in the literature, where at first the volume loss of the solvent and the volume shrinkage of the structure is equal.^21^ Near the cutoff point, at the point of maximum shrinkage, a sharp drop is noticed for the fractal dimension, which was not observed for the UN sample. Once again, this could be because the samples were fully dense and the fractal structure consisted of a combination of the silica structure and the hexane, which would explain the drastically lower overall relative density of the system. However, the UN sample shows a different behavior and almost no decrease in the fractal dimension, which leads to believe that this system cannot be interpreted as a composition of both the silica and hexane contributions. Since water condensation of two silanol (Si–OH) groups is likely to occur for this specimen,^15^ the additional water could potentially be adsorbed at the surface of the material, creating a layer between silica and hexane, inhibiting the sudden decrease in fractal dimension. After the cutoff point, all samples increased in fractal dimension, although being at their point of maximum shrinkage. This supports the theory previously discussed, that the fractal system consists of the silica network and a layer of solvent with air being the contrast. Therefore, the increase in relative density might be explained by a loss of solvent, rather than the shrinkage of the material. Lastly, the decrease in the fractal dimension was pronounced for the modified HM, TE, and TM samples. This was a clear indication of irreversible densification due to condensation reactions for the UN sample, resulting in a higher relative density. Surprisingly, the HM and TE samples which also experienced substantial irreversible shrinkage, did not show this correlation. However, since the previously described correlation length decreased significantly, the network likely recovered its fractal structure, but over a significantly lower order of size. It is suggested that the interpretation of the relative density might only be feasible with a contribution of adsorbed solvent and should only be applied, when the samples were completely dried. At the point of a fully dried material, it might also be used for the evaluation of the initial aggregation behavior if the sample did not show irreversible shrinkage.

Finally, the radius of primary particles, as evaluated from the scattering model is shown in Fig. 3G. In a monodisperse system, this primary particle radius can be seen as an oscillation of the X-ray scattering profile. The absence of this oscillation is evidence of polydispersity, which is reasonable in this sample system.^47^ A lognormal distribution was assumed for single- and multi-core iron oxide particles in the literature,^55^ whereas a monomodal distribution was assumed in other work.^9^ Since the polydispersity also affected the curvature of the SAXS profile, a value of 0.5 was set as a lognormal polydispersity for all calculations to improve the comparability of the samples.

At the first stage (I), values of 4.5 Å, 3.5 Å, 4.1 Å, and 3.8 Å were observed for the radius of primary particles for the UN, HM, TE, and TM samples, respectively. Once more, this indicated that the UN sample experienced aging, which reportedly resulted in bigger primary particles.^48^ The difference between the HM, TE, and TM samples could be uncertainty. On the contrary, it could display the change in size due to the silyl group, where the triethylsilyl groups of the TE sample should be bigger than the trimethylsilyl groups of the HM, and TM samples. The fully dried samples (IV) ended up at 4.5 Å (UN), 4.6 Å (HM), 5.0 Å (TE), and 5.2 Å (TM). These values differed slightly from a previous study though showing the same trends, which may be explained by the use of a different scattering model and a more robust fitting algorithm for this publication.^42^ Overall these results were in the scope of reported values in the past.^12^ The evolution of the two-phase system (II) showed an overall decrease in primary particle size, while the decline was similar for the modified HM, TE, and TM samples reaching roughly values of 3.4, 3.8, and 3.4 at the cutoff point (II/III), whereas the UN sample showed a more drastic decrease, ending up at 3.2. Afterwards for the three-phase system (III), the primary particle size increased again. Similarly, the fiber diameter in hydrogels investigated by small-angle neutron scattering was also reported in the literature to increase during drying.^56^ The slopes of the primary particle size evolution of the samples were very similar, reaching a plateau that was constant throughout the remaining measurement time, ending at the values of the dried samples.

Previous studies have shown that the radius of the primary particles is likely independent of the fractal dimension.^9^ However, the primary particle radius is also the cutoff distance for the fractal dimension at high *Q*, where smaller objects are not fractal anymore. While the initial decline in the size of the primary particles may be interpreted as a contraction of the elementary units, it was surprising that the UN sample seemingly shrunk more. This could be indicative of a strengthening of the modified samples, therefore withstanding the shrinkage. Likewise, the surface silylation could introduce some steric hindrance, potentially hindering the particles' contraction. After the maximum shrinkage, all samples increased in primary particle size. Noticeable was that the UN sample recovered its initial values. This is a strong indication that even without surface modification, some relaxation on a molecular level can be observed. On the other hand, the HM, TE, and TM samples increased to even higher values compared to the start of the measurements. One explanation might be a loss in the fractal region at the near molecular level. Due to a reorganization of the network, they might lose part of their fractal structure at a very small scale, shifting the cutoff point of the primary particles to lower *Q*.

## 3. Conclusions

**Fig. 4:**
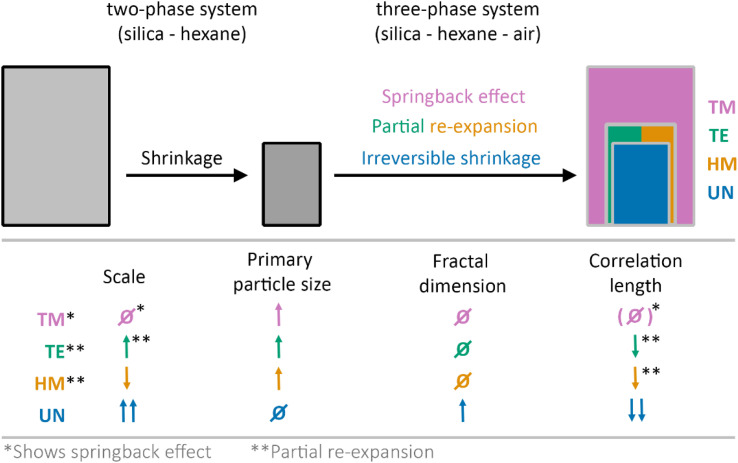
Schematic of drying gels that show irreversible shrinkage, partial re-expansion, or the full springback effect of unmodified UN (blue), hexamethyldisilazane-modified HM (orange), triethylchlorosilane-modified TE (green), and trimethylchlorosilane-modified TM (magenta) samples. During the experiment, a change from a two-phase system (silica-hexane) to a three-phase system (silica-hexane-air) was assumed. Tendencies of the change from first to last measurement point of the scale, primary particle size, fractal dimension and correlation length are given. In this regard, ↓ and ↑ represent lower or higher final values, respectively. *Ø* constitutes roughly the same starting and end values. Additionally, an asterisk (*) is depicting samples or parameters that correlated with the SBE or partial re-expansion (**). For simplification, it was assumed that the samples did not experience drying before the first measurement.

In this work, the drying of unmodified (UN), hexamethyldisilizane-modified (HM), triethylchlorosilane-modified (TE), and trimethylchlorosilane-modified (TM) silica gel monoliths was investigated *in situ* using synchrotron X-ray scattering coupled with optical imaging. The X-ray scattering data were evaluated considering contributions of the hexane and the fractal silica network, allowing in this way for determining the structural parameters at each drying stage. A change from a two-phase system of silica and hexane to a three-phase system of silica, hexane and air was assumed and confirmed by the experiments. While the TM sample showed the SBE, the HM and TE samples experienced only partial re-expansion, and the UN sample shrunk irreversibly. This is schematically depicted in Fig. 4 with an additional summary of structural parameters comparing the starting and final drying stages.

The evolution of the primary particle size unravelled differences between the modified and the unmodified samples. While there might be some reorganization of the primary particles within the modified samples, the SBE is likely not induced by the change on the near molecular level, as even the UN sample showed some relaxation at this scale (Fig. 4). Moreover, the SBE, partial re-expansion, or irreversible shrinkage was observed in the evolution of the correlation length, which is in the size range of individual clusters. Here, an increase in size of the correlation length directly correlated with a re-expansion of the sample.

The correlation length and the fractal dimension indicated that the gel network might experience densification near the point of maximum shrinkage, where the sample becomes fully compact. Furthermore, the fractal dimension of the material was also affected by the shrinkage and re-expansion of the material. In contrast to an assumption made in a previous publication, where the recovery of the fractal dimension from the first to last point might refer to full re-expansion of the material,^41^ here the modified samples with different degrees of re-expansion showed similarities in their fractal dimension values. While this might be explained by a cracking of the material that was observed in the digital photographs, it could also indicate that the fractal dimension should not be solely considered for evaluating the SBE, but rather in conjunction with the correlation length. In this regard, the influence of solvent adsorption on the surface of the samples must be considered as well for the interpretation of the fractal dimension and correlation length.

It was shown that the transmission and the scaling factor of the fractal contribution in the SAXS region were sensitive to the shrinkage and re-expansion during the SBE (Fig. 4). Especially the latter could be used to qualitatively describe the volume change while pointing out the formation of cracks or voids inside the material. Furthermore, the determined hexane content confirmed a slower drying rate of the modified samples in comparison to the unmodified specimen, which is in line with our previous results and is explained by a different affinity of the solvent with the samples' surface, and is in conjunction with a previous study.^41^ This extension of the drying period might be a supporting factor for the SBE.

While the nucleation of a supramolecular gel was investigated *in situ* in the literature,^57^ for APD aerogels, the drying is the crucial step. We have shown in our previous study that some structural features of the shrinkage and re-expansion can be investigated *via* X-ray scattering.^41^ Here, we have shown for samples with different silylation that this evaluation can be enhanced severely by applying a model with different intensity contributions for the synchrotron X-ray dataset. Information about various structural parameters in a range of roughly 4.5 Å to 100 Å in size was obtained. Moreover, the evaluation of the scattering model gives nano-structural insights (*e.g.*, primary particle size, correlation length) that would otherwise not be easily accessible. A fundamental understanding of drying is needed to produce monolithic APD aerogels, which this study could contribute to.

## 4. Materials and methods

### 4.1. Synthesis of silica gels

The samples were produced following an adaptation of Wei *et al.* with different silylation agents.^58^ Exemplary, 10.4 g of tetraethyl-orthosilicate (Alfa Aesar, ≥99%) was mixed with 4.4 mL of ethanol (Carl Roth, >99.5%, Ph.Eur., reinst), 4.4 mL of a mixture (105 μL in 438.19 mL) of hydrochloric acid (Sigma-Aldrich, Merck, 37%) and ethanol, as well as 0.9 mL of deionized water (DIW) and stirred for 90 min. Additional 14.6 mL of ethanol, and 2.4 mL of a mixture (1 g in 168 g) of ammonium hydroxide (Sigma-Aldrich, Merck, 25%) and DIW were added to the solution, stirred for 30 min and afterwards left to gel in smaller cuboid Teflon molds (1.5 cm/1 cm/0.6 cm). These cuboid samples were aged for 24 h at 50 °C. This resulted in roughly 3 g of Silica, which was 9.3 wt% of the final gel.

Afterwards, the gels were washed at room temperature for at least 24 h with an excess amount of ethanol (VWR, ≥96% denatured, GPR RECTAPUR®), as well as mixtures of 25 vol%/75 vol%, 50 vol%/50 vol%, 75 vol%/25 vol% of hexane (Carl Roth, *n*-hexane, >99%) and ethanol, and finally four times with pure hexane. While the unmodified sample (denoted as UN) was stored in the hexane, the surface-modified samples were treated further, as reported previously,^42^ with either trimethylchlorosilane (Sigma-Aldrich, Merck, TMCS, purified by redistillation >99%), triethylchlorosilane (Sigma-Aldrich, Merck, TECS, 99%), or hexamethyldisilazane (Sigma-Aldrich, Merck, HMDS, reagent grade ≥99%), denoted as TM, TE, and HM, respectively. For this purpose, four individual solvent exchanges were conducted under equal conditions with twice an excess of 3 vol%, and 6 vol% mixtures of silylation agent in hexane. Finally, these samples were rinsed four times with hexane, and stored in it until the synchrotron experiments were conducted.

### 4.2. *In situ* X-ray scattering measurements and data integration

**Fig. 5:**
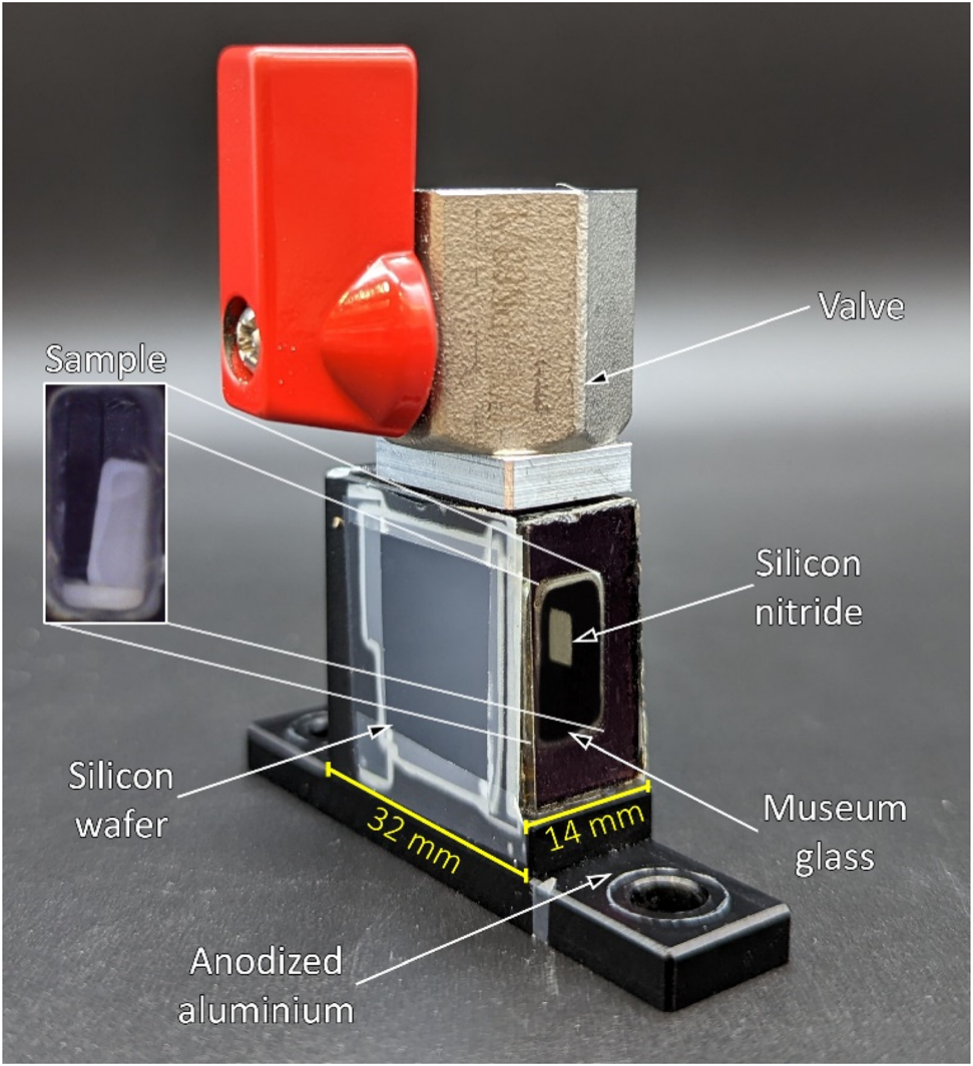
Photograph of the *in situ* measurement cell consisting of anodized aluminium, a valve on the top, a museum glass in-front, and a silicon wafer and silicon nitride window in direction of the X-ray beam. An exemplary photograph of a gel sample inside the measurement cell is shown on the left side. The scale bars (yellow) of the width and depth are given.

The X-ray scattering measurements were performed on the samples, which were stored in hexane. The soaked samples were investigated at the BESSY II synchrotron of the Helmholtz Zentrum für Materialien und Energie (Germany, Berlin) at the μSpot beamline of the Max Planck Institute of Colloids and Interfaces.^59^ Three individual measurement cells were attached to a stage to allow for parallel investigations. These measurement cells were constructed from anodized aluminium and sealed off in direction of the X-ray beam with a silicon wafer and silicon nitride window (NORCADA Low stress SiNx Membrane, 10 mm length/width, 1000 nm thickness), on top with a valve (1/8′′, PN63/1.4408, shortened with adapter to *ca.* 26 mm), and in the front with a museum glass as shown in Fig. 5. A digital microscope camera (TOOLKRAFT USB microscope, 5 MP) was focused on the sample through the museum glass to capture images over the course of the X-ray scattering measurements. At the time of a measurement, two samples were transferred into individual measurement cells, whereas an empty cell was used for background correction. The valves were opened fully prior to the measurement. With an exposure of 10 s, the stage was alternating between one measurement for the empty cell, and three measurements for the two measurement cells, capturing three different positions (Pos. 1–3) with 1 mm height distance from each other. Because of the shrinkage and (partial) re-expansion of the samples, the investigated volume changed throughout the experiment. The synchrotron beam had an energy of 15 keV, using a B4C/Mo Multilayer (2 nm period) monochromator. A spot size of 30 × 30 μm^2^ was adjusted by a series of pinholes. The scattering data was collected using an Eiger 9M detector with a 75 × 75 μm^2^ pixel size. At the same distance of the samples, a quartz reference was fixed. It was used to determine the sample to detector distance, beam center, tilt, and rotation. A glassy carbon Standard Reference Material 3600 (SRM 3600) of the National Institute of Standards and Technology (NIST) was measured for absolute intensity calibration.^60^ The intensity was not calibrated for the change in sample size over time. The captured data was processed using the directly programmable data analysis kit (DPDAK).^61^ The data was normalized over the intensity of the primary beam, and the measured background of the empty cell was subtracted. The X-ray scattering measurements were radially integrated, providing the scattered intensity *I*(*Q*) as a function of the momentum transfer *Q*, using the wavelength of the synchrotron beam λ and the scattering angle *θ*:1
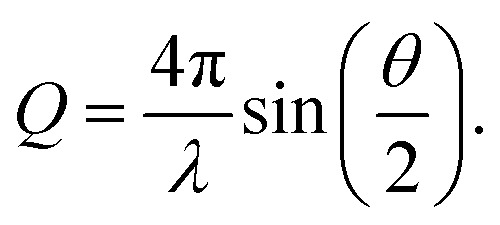


This translated to a *Q* range of *ca.* 0.007 Å^−1^ to 4 Å^−1^. An in-house python script was applied on the normalized data set to estimate the uncertainty, as well as absolute normalization using the SRM 3600. The latter was applied, following the recommendations of NIST.^60^ A plot of the measured standard and the provided standardized data is shown in Fig. S15.† The processed X-ray scattering data, as well as the captured photographs throughout the experiment are accessible from Note S6.†

### 4.3. Structure and form factor analysis

SasView v5.0.5 (http://www.sasview.org/, Accessed 14.03.2023) with its python library ‘sasmodels’ was used to evaluate structural features of the samples, the size of primary particles, their cluster size and fractal dimension, as well as the scale of the fractal structure, and the hexane and silica peaks in the WAXS region. Here, it was assumed that the overall scattered intensity of the specimen *I*_total_ consisted of spherical particles in a fractal structure *I*_fractal_, a hexane contribution *I*_hexane_, and peaks of the silica backbone *I*_silica_:

2 *I*_total_ = *I*_fractal_ + *I*_silica_1_ + *I*_silica_2_ + *I*_hexane_

The sum of the different intensity contributions with an overlaid measurement point is shown in Fig. S16.†

The scattering intensity contributions of the hexane, as well as the peaks were evaluated by assuming a Lorentzian peak (“peak_lorentz”), as taken from the SasView User Documentation (https://www.sasview.org/docs/user/models/peak_lorentz.html, Accessed 14.03.2023), which consists of a scaling factor *s*, a constant background *C*, a half-width at half-maximum (HWHM) *H*, and a peak position *Q*_0_:3
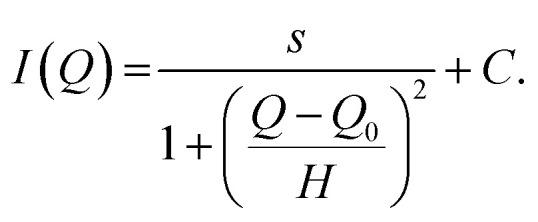


The scattering intensity contribution of the spherical particles with fractal structure *I*_fractal_ was determined by a “fractal” model as taken from the SasView User Documentation (https://www.sasview.org/docs/user/models/fractal.html, Accessed 14.03.2023). This structure was originally reported by Teixeira *et al.*^46^ The radially integrated scattering data was described by:

4 *I*(*Q*)_fractal_ = *αϕV*_block_(*ρ*_block_ − *ρ*_solvent_)^2^*P*(*Q*)*S*(*Q*) + *C*_fractal_

5 *P*(*Q*) = *F*(*QR*_0_)^2^.

6
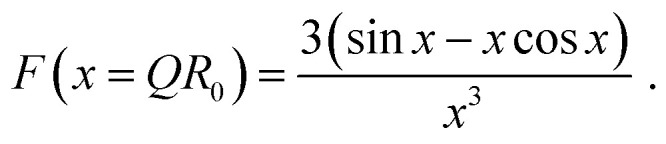
7
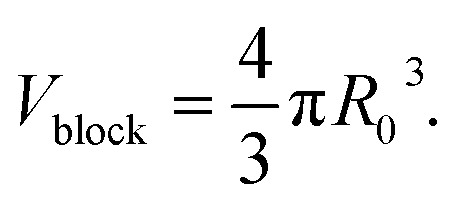
8
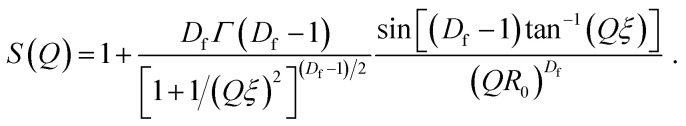


As shown in eqn (4), *I*(*Q*)_fractal_ was a function of the volume fraction *ϕ*, which is the ratio of the solid backbone and the solvent. Additionally, *α* is given as a free parameter proportional to the volume fraction, allowing to evaluate the scattering data not normalized for the sample width. The volume of the building block *V*_block_, as well as the scattering length density (SLD) of the solid backbone *ρ*_block_, referred to the surface modified solid silica backbone. On the other hand, the SLD of the solvent *ρ*_solvent_ consisted of either pure hexane, a mixture of hexane and air, or pure air for the beginning, intermediate or end of the experiment, respectively. Furthermore, the fractal scattering contribution comprised of a form factor *P*(*Q*), a structure factor *S*(*Q*), and a background *C*_fractal_. The former inherits the size of primary particles *R*_0_ (eqn (5)), and is a function of the averaged assembly of particles (eqn (6)). The structure factor *S*(*Q*) of eqn (4) and (8) consisted of the Gamma function *Γ*, the fractal dimension *D*_f_, the radius of primary particles *R*_0_, and the correlation length commonly referred to the cluster size *ξ*. A detailed description of the combination of these intensity contributions in SasView can be found in Note S7.†

The data set with its d*I* error and d*Q* smearing was used. The following assumptions were made: (i) for each sample, the hexane content was 100 vol% at the first measurement; (ii) the sample started to shrink and the hexane content evaporated proportionally; (iii) at the point where the specimen was completely shrunken, there was still hexane evaporation, leading to a mixture of air and hexane; (iv) at the last measurement, the hexane content was 0 vol%; (v) the change of the SLD of the silica backbone was negligible; (vi) the position of the silica and hexane peaks did not change throughout the experiment; (vii) the intensity contribution of the silica peaks was coupled to the scale and to this effect proportional to the volume fraction of the sample; (viii) the lognormal polydispersity of the primary particles did not change within the experiment, assuming that clusters and primary particles changed proportionally; (ix) there are no drastic changes from one data point to the next, making a batch chain fit reasonable. An in-depth description of these assumptions can be found in Note S8.†

Throughout the experiment, the parameters were limited by using values reported in literature and previous work.^12,41^ These limitations are documented in Note S9.† Furthermore, a schematic of how the scattering model was applied for each sample can be found in Fig. S17.† The last measurement point was loaded, assuming that no hexane was left inside samples and the background, scale of the fractal contribution, radius of primary particles, fractal dimension, correlation length, as well as the scales, HWHM and position of the (modified) silica peaks was fitted. The input and output values of this fit can be seen in Tables S1 and S2.† Afterwards, the first measurement point was loaded with the calculated values of the previous output. The position and HWHM of the silica peaks were kept constant and the scale was constrained to the scale of the fractal contribution. Additionally, the hexane scale was fitted. The input and output of these fits of the first measurements can be seen in Tables S3 and S4.† Then the output of this fit was used for the batch fits, which fitted the same parameters as before, limiting the hexane scale to the determined value. The entire dataset of the individual sample was split at the point where a two-phase system of silica-hexane was assumed to become a three-phase system of silica-hexane-air. This transition point was determined with the digital photographs where the samples showed a noticeable change in transparency. While the first half of the batch considered hexane for the SLD of the solvent, for the second half it was constrained to the scale of hexane. The batch was calculated in chain, always considering the output of the prior measurement. The initial input of these batch ranges is shown in Tables S5 and Table S6.†

## Author contributions

F. Z.: conceptualization, methodology, software, formal analysis, investigation, data curation, writing – original draft, writing – review & editing, visualization, project administration; E. S.: conceptualization, methodology, software, validation, formal analysis, investigation, data curation, writing – original draft, writing – review & editing; U. S.: validation, writing – review & editing, supervision; M. F. B.: validation, writing – review & editing, supervision; W. W.: conceptualization, validation, resources, writing – original draft, writing – review & editing, project administration, funding acquisition; A. G.: conceptualization, writing – review & editing, supervision, project administration, funding acquisition.

## Conflicts of interest

There are no conflicts of interest to declare.

## Supplementary Material

NA-006-D3NA00584D-s001
