# Supplementary material for: Springback effect of ambient-pressure-dried silica aerogels: nanoscopic effects of silylation revealed by in situ synchrotron X-ray scattering
Source: Nanoscale Adv. 2023 Nov 28;6(1):111–25. doi: 10.1039/d3na00584d (PMC10729877; doi:10.1039/d3na00584d)
Supplement: NA-006-D3NA00584D-s001 [file NA-006-D3NA00584D-s001.pdf]

Electronic Supplementary Material (ESI) for Nanoscale Advances.
This journal is © The Royal Society of Chemistry 2023

Supporting information

Springback effect of ambient-pressure-dried silica aerogels: Nanoscopic effects of silylation revealed by in-situ synchrotron X-ray scattering

Electronic Supplementary Information

Table of contents:

1 **Fig. S1.** In-situ SAXS diagrams of the unmodified UN (A), hexamethyldisilazane-modified HM (B), triethylchlorosilane-modified TE (C), and trimethylchlorosilane-modified TM (D) samples over the duration of the drying.

2 **Fig. S2.** SAXS measurements of a hexane capillary (pink), an exemplary sample at the start (first measurement, blue) and end (last measurement, cyan), as well as the subtracted intensities of first measurement and hexane (red), as well as first and last measurement (grey) are shown.

3 **Note S1.** Calculation of the hexane content, fractal and Porod slopes

4 **Fig. S3.** The percentage of hexane of the unmodified UN (blue), hexamethyldisilazane-modified HM (orange), triethylchlorosilane-modified TE (green), and trimethylchlorosilane-modified TM (magenta) samples over the duration of the synchrotron X-ray scattering experiments. The intensity was calculated by fitting two Lorentzian peaks, with one referring to the silica backbone and one to the hexane contribution, and using the normalized area of the hexane peak for the evaluation. This approach was described elsewhere in detail.

5 **Fig. S4.** Reduced In-situ SAXS diagrams of the unmodified UN (A), hexamethyldisilazane-modified HM (B), triethylchlorosilane-modified TE (C), and trimethylchlorosilane-modified TM (D) samples over the duration of the drying. The contribution of hexane was subtracted by using the scaling of the Lorentzian peak evaluation (Fig. S3). The position of the hexane peak was taken from the measurement of pure hexane inside a glass-capillary (Fig. S2). This approach was described elsewhere in detail.

6 **Note S2.** Results and discussion of the Porod slope

7 **Fig. S5.** The Porod slope of Pos. 2 of the unmodified UN (blue), hexamethyldisilazane-modified HM (orange), triethylchlorosilane-modified TE (green), and trimethylchlorosilane-modified TM (magenta) samples over the duration of the drying of calculated from the Q range of 0.31 Å-1 to 0.45 Å-1 for the measurements of Fig. S1 (A), and the hexane reduced data of Fig. S4 (B). The calculations are described in detail elsewhere.

8 **Note S3.** Fractal slope evaluation

9 **Fig. S6.** The Fractal slope of Pos. 2 of the unmodified UN (blue), hexamethyldisilazane-modified HM (orange), triethylchlorosilane-modified TE (green), and trimethylchlorosilane-modified TM (magenta) samples over the duration of the drying of calculated from the Q range of 0.03 Å-1 to 0.24 Å-1 for the measurements of Fig. S1 (A), and the hexane reduced data of Fig. S4 (B). The calculations are described in detail elsewhere.

10 **Note S4.** Supplementary results and discussions of scattering model parameters

11 **Note S5.** Goodness of the scattering model evaluation

12 **Fig. S7.** The scattering model evaluation of the synchrotron X-ray scattering measurements of Pos. 2 of the unmodified UN (blue, circle), hexamethyldisilazane-modified HM (orange, triangle), triethylchlorosilane-modified TE (green, square), and trimethylchlorosilane-modified TM (magenta, diamond) samples with their corresponding standard deviation (bar line) are shown for the duration of the experiment. Additionally, dashed vertical lines in the color of the appropiate samples, show the estimated crossover of the two-phase system (silica-hexane) to a three-phase system (silica-hexane-air). The Chi-squared error (**A**) shows the goodness of the fit. The scale of the hexane peak (B) shows the evaporation. The scattering length density of the solvent (D) was constrained to the hexane peak in the three-phase system. The peaks of the (modified) silica backbone (C, E) were directly constrained to the scale/volume fraction of the samples. The background (F) and the scale of the fractal contribution (G) were left unrestricted.

13 **Fig. S8.** The SasView evaluation of the Pos. 1 synchrotron X-ray scattering measurements of the unmodified UN (blue, circle), hexamethyldisilazane-modified HM (orange, triangle), triethylchlorosilane-modified TE (green, square), and trimethylchlorosilane-modified TM (magenta, diamond) samples with their corresponding standard deviation (bar line) are shown for the duration of the experiment, as well as the measured transmission of the samples (A). Additionally, dashed vertical lines in the color of the appropiate samples, show the estimated crossover of the two-phase system (silica-hexane) to a three-phase system (silica-hexane-air). The Chi-squared error (B) shows the goodness of the fit. The scale of the hexane peak (C) correlated with the scattering length density of the solvent (D), which was pure hexane at first, and was constrained to the hexane peak in the three-phase system. The background (E) and the scale of the fractal contribution (F) were left unrestricted. The correlation length (G), fractal dimension (H) and the primary particle radius (I) were evaluated from the fractal intensity contributions of the samples.

14 **Fig. S9.** The SasView evaluation of the Pos. 3 synchrotron X-ray scattering measurements of the unmodified UN (blue, circle), hexamethyldisilazane-modified HM (orange, triangle), triethylchlorosilane-modified TE (green, square), and trimethylchlorosilane-modified TM (magenta, diamond) samples with their corresponding standard deviation (bar line) are shown for the duration of the experiment, as well as the measured transmission of the samples (A). Additionally, dashed vertical lines in the color of the appropiate samples, show the estimated crossover of the two-phase system (silica-hexane) to a three-phase system (silica-hexane-air). The Chi-squared error (B) shows the goodness of the fit. The scale of the hexane peak (C) correlated with the scattering length density of the solvent (D), which was pure hexane at first, and was constrained to the hexane peak in the three-phase system. The background (E) and the scale of the fractal contribution (F) were left unrestricted. The correlation length (G), fractal dimension (H) and the primary particle radius (I) were evaluated from the fractal intensity contributions of the samples.

15 **Fig. S10.** The synchrotron X-ray scattering measurement dataset (straight line) compared to the SasView evaluation (dashed line) of Pos. 1 of A-D) the first measurement (I), E-H) one exemplary measurement of the two-phase system (II), I-L) one exemplary measurement of the three-phase system (III), M-P) the last measurement (IV). The goodness of the fit is shown for the unmodified UN (A, E, I, M), hexamethyldisilazane-modified HM (B, F, J, N), triethylchlorosilane-modified TE (C, G, K, O), and trimethylchlorosilane-modified TM (D, H, L, P) samples.

16 **Fig. S11.** The synchrotron X-ray scattering measurement dataset (straight line) compared to the SasView evaluation (dashed line) of Pos. 2 of A-D) the first measurement (I), E-H) one exemplary measurement of the two-phase system (II), I-L) one exemplary measurement of the three-phase system (III), M-P) the last measurement (IV). The goodness of the fit is shown for the unmodified UN (A, E, I, M), hexamethyldisilazane-modified HM (B, F, J, N), triethylchlorosilane-modified TE (C, G, K, O), and trimethylchlorosilane-modified TM (D, H, L, P) samples.

17 **Fig. S12.** The synchrotron X-ray scattering measurement dataset (straight line) compared to the SasView evaluation (dashed line) of Pos. 3 of A-D) the first measurement (I), E-H) one exemplary measurement of the two-phase system (II), I-L) one exemplary measurement of the three-phase system (III), M-P) the last measurement (IV). The goodness of the fit is shown for the unmodified UN (A, E, I, M), hexamethyldisilazane-modified HM (B, F, J, N), triethylchlorosilane-modified TE (C, G, K, O), and trimethylchlorosilane-modified TM (D, H, L, P) samples.

18 **Fig. S13.** The normalized residuals of the SasView evaluation calculated by the difference of the model and measurement data and divided by the error of the measurement of the unmodified UN (blue), hexamethyldisilazane-modified HM (orange), triethylchlorosilane-modified TE (green), and trimethylchlorosilane-modified TM (magenta) samples. The three positions were plotted for A-C) the first measurement (I), D-F) one exemplary measurement of the two-phase system (II), G-I) one exemplary measurement of the three-phase system (III), and J-L) the last measurement (IV).

19 **Fig. S14.** Comparison of drying velocity for the UN (blue) and TM (magenta) samples. A) Time-dependant hexane intensity evolution (blue) and modelling (green) for the UN sample. B) Time-dependant hexane intensity evolution (magenta) and modelling (green) for the TM sample. C) Comparison between hexane content model derivatives for the UN (blue) and TM (magenta) samples. It can be observed that for the first 4 hours TM sample dries faster than UN. D) The UN/TM drying velocity ratio is less equal one for 4.08 h. Above this time, the drying velocity of the UN is faster than the TM sample, which is represented by values greater than one.

20 **Fig. S15.** Reported Standard reference material 3600 (red) of the National Institute of Standards and Technology (NIST) overlaid with a measurement of this standard (blue) for absolute intensity calibration.

21 **Note S6.** DOI of processed data

22 **Fig. S16.** Exemplary X-ray scattering measurement (blue) overlapped with the different intensity contributions, namely a fractal contribution (orange) and three Lorentzian peaks related to the hexane (green), as well as the silica backbone (red/magenta).

23 **Fig. S17.** Schematic of the procedure of the SasView evaluation: The measurement datasets are loaded, and the last measurement selected to determine the scale, half-width half-maximum (HWHM) of the silica peaks in the wide-angle X-ray scattering (WAXS) region. Afterwards, the first measurement is used to assess the initial hexane scale in the WAXS region. The cutoff point of the two-phase (silica-hexane) to the three-phase system (silica-hexane-air) was determined by evaluating the photographs. The two ranges of measurements were used for the batch evaluation.

24 **Table S1.** SasView evaluation input values of the last measurement points of the three positions for the unmodified UN, hexamethyldisilazane-modified HM, triethylchlorosilane-modified TE, and trimethylchlorosilane-modified TM samples. The fitting algorithm DREAM was used (--burn=100 --pop=10 --init=eps --thin=1 --steps=0), and a lognormal polydispersity was assumed for the radius of primary particles. (A_radius_pd.value = 0.5, A_radius_pd_n.value = 80, A_radius_pd_nsigma.value = 8). The background, scale of the fractal contribution, correlation length, radius, the two scales of the silica contributions, as well as their Half-width half-maximum (HWHM) and peak positions were fitted within the documented ranges.

25 **Table S2.** SasView evaluation output values of the last measurement points of the three positions for the unmodified UN, hexamethyldisilazane-modified HM, triethylchlorosilane-modified TE, and trimethylchlorosilane-modified TM samples. The fitted values are shown with their respective 95 % confidence intervals, rounded to the 2nd digit (4th for the background). Additionally, the goodness of the fit is shown by the Chi-square (Chi2) values.

26 **Table S3.** SasView evaluation input values of the first measurement points of the three positions for the unmodified UN, hexamethyldisilazane-modified HM, triethylchlorosilane-modified TE, and trimethylchlorosilane-modified TM samples. The fitting algorithm DREAM was used (--burn=100 --pop=10 --init=eps --thin=1 --steps=0), and a lognormal polydispersity was assumed for the radius of primary particles. (A_radius_pd.value = 0.5, A_radius_pd_n.value = 80, A_radius_pd_nsigma.value = 8). The background, scale of the fractal contribution, correlation length, radius, the scale of the hexane contribution were fitted within the documented ranges.

27 **Table S4.** SasView evaluation output values of the first measurement points of the three positions for the unmodified UN, hexamethyldisilazane-modified HM, triethylchlorosilane-modified TE, and trimethylchlorosilane-modified TM samples. The fitted values are shown with their respective 95 % confidence intervals, rounded to the 2nd digit (4th for the background). Additionally, the goodness of the fit is shown by the Chi-square (Chi2) values.

28 **Table S5.** SasView evaluation input values of the first batch measurements of the three positions for the unmodified UN, hexamethyldisilazane-modified HM, triethylchlorosilane-modified TE, and trimethylchlorosilane-modified TM samples. The fitting algorithm DREAM was used (--burn=100 --pop=10 --init=eps --thin=1 --steps=0), and a lognormal polydispersity was assumed for the

This journal is © The Royal Society of Chemistry 2023

radius of primary particles. (A_radius_pd.value = 0.5, A_radius_pd_n.value = 80, A_radius_pd_nsigma.value = 8). The background, scale of the fractal contribution, correlation length, radius, and the scale of the hexane contribution were fitted within the documented ranges. A chain batch fit and therefore the previous measurement outputs were used.

29 **Table S6.** SasView evaluation input values of the first batch measurements of the three positions for the unmodified UN, hexamethyldisilazane-modified HM, triethylchlorosilane-modified TE, and trimethylchlorosilane-modified TM samples. The fitting algorithm DREAM was used (--burn=100 --pop=10 --init=eps --thin=1 --steps=0), and a lognormal polydispersity was assumed for the radius of primary particles. (A_radius_pd.value = 0.5, A_radius_pd_n.value = 80, A_radius_pd_nsigma.value = 8). The background, scale of the fractal contribution, correlation length, radius, the scale of the hexane contribution were fitted within the documented ranges. A chain batch fit and therefore the previous measurement outputs were used. The values of the B_scale were cut off after the 2nd digit, although the full number was used for the input.

30 **Note S7.** Creating a custom SasView model

31 **Note S8.** Assumptions made in the modelling of SasView parameters

32 **Note S9.** Restrictions of the parameters in the SasView model

33 **References for the supporting information**

Supporting information

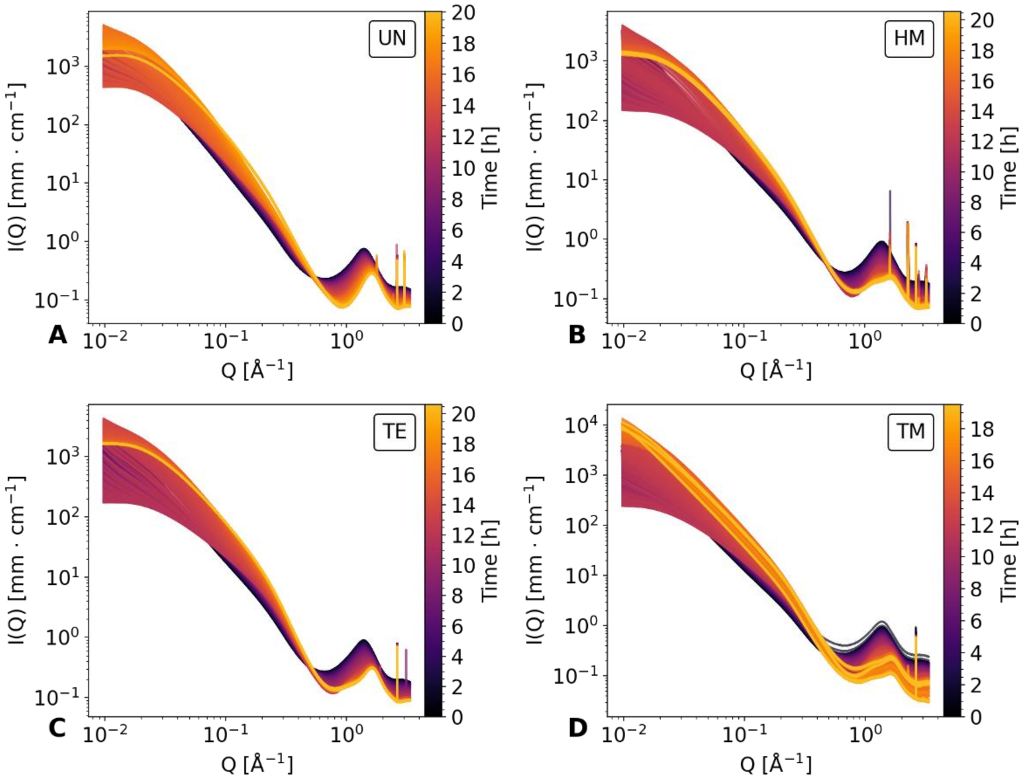

Fig. S1. In-situ SAXS diagrams of the unmodified UN (**A**), hexamethyldisilazane-modified HM (**B**), triethylchlorosilane-modified TE (**C**), and trimethylchlorosilane-modified TM (**D**) samples over the duration of the drying.

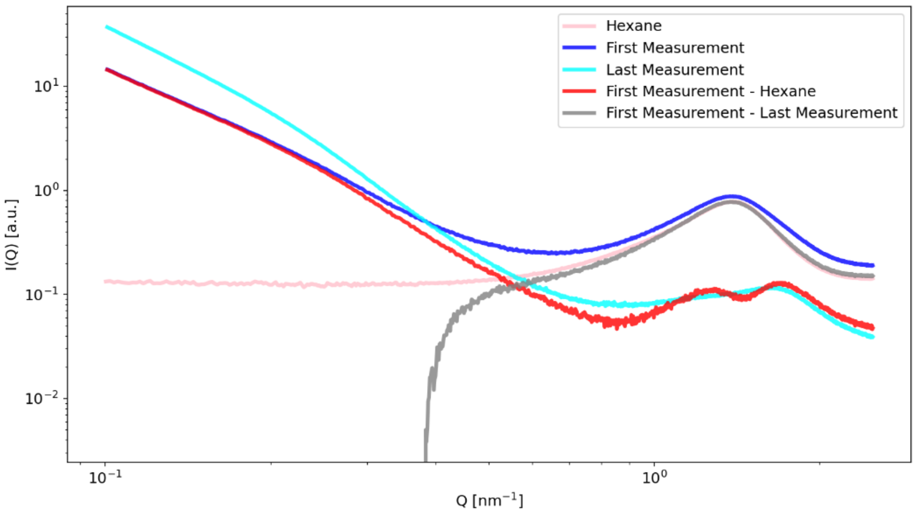

Fig. S2. SAXS measurements of a hexane capillary (pink), an exemplary sample at the start (first measurement, blue) and end (last measurement, cyan), as well as the subtracted intensities of first measurement and hexane (red), as well as first and last measurement (grey) are shown. The hexane measurement was reported elsewhere.[1]

Note S1. Calculation of the hexane content, fractal and Porod slopes

The calculations of previous work using Lorentz peak areas in the wide-angle region to determine the hexane content was replicated for the current dataset.[1] The DPDAK Peak Fit plugin was used to determine the Lorentz peak areas.[2] The evaporation of the hexane content is shown in Fig. S3. Generally, the intensity was declining monotonously but showed a high error for the very low hexane intensities. Information about the hexane content were used to subtract this contribution from the X-ray scattering dataset, consistently to previous work,[1] which is shown in Fig. S4. Since this will not be a focus of this study, it will not be discussed extensively. This approach enhances the visibility of the two peaks of the silica backbone otherwise overshadowed by the hexane contribution. Pythons scipy stats library was used for linear regression and calculating of the Fractal and Porod slopes.[3] These two calculations inherited statistical errors directly linked to the fit.

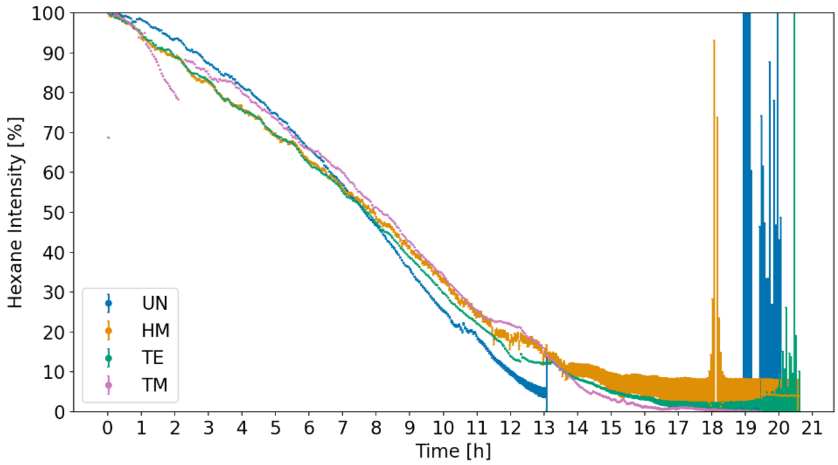

Fig. S3. The percentage of hexane of the unmodified UN (blue), hexamethyldisilazane-modified HM (orange), triethylchlorosilane-modified TE (green), and trimethylchlorosilane-modified TM (magenta) samples over the duration of the synchrotron X-ray scattering experiments. The intensity was calculated by fitting two Lorentzian peaks, with one referring to the silica backbone and one to the hexane contribution, and using the normalized area of the hexane peak for the evaluation. This approach was described elsewhere in detail.[1]

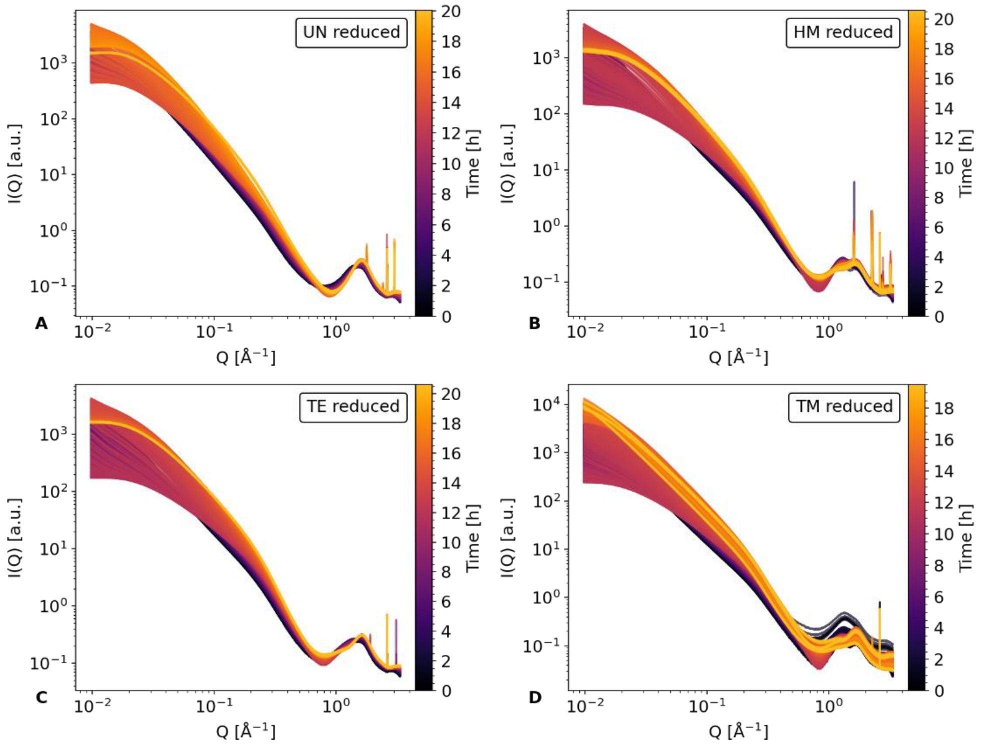

Fig. S4. Reduced In-situ SAXS diagrams of the unmodified UN (**A**), hexamethyldisilazane-modified HM (**B**), triethylchlorosilane-modified TE (**C**), and trimethylchlorosilane-modified TM (**D**) samples over the duration of the drying. The contribution of hexane was subtracted by using the scaling of the Lorentzian peak evaluation (Fig. S3). The position of the hexane peak was taken from the measurement of pure hexane inside a glass-capillary (Fig. S2). This approach was described elsewhere in detail.[1]

Note S2. Results and discussion of the Porod slope

The linear regression analysis of the Porod slope was replicated from a previous study for comparison,[1] and is shown in Fig. S5. The slope evolution shows a split into two ranges, where first a small decrease is seen for all samples, followed by a sharp decline. This decrease was reported in literature, where the dried gels showed a slope of -4 in contrast to a solvent-filled specimen with -3.5.[4] While the UN, and TM samples ended at roughly -4, the HM showed slightly higher values, and the TE sample a Porod slope of ca. -4.3. This result would require more investigation but was not a focus of this study. While a Porod slope of 4 refers to a sharp interface, and values of 3 could be indicative of a rough surface, values between the two could be another mass fractal.[5] Similarly to the calculations of the Porod slope, the analysis of the fractal slope is shown in Fig. S6.

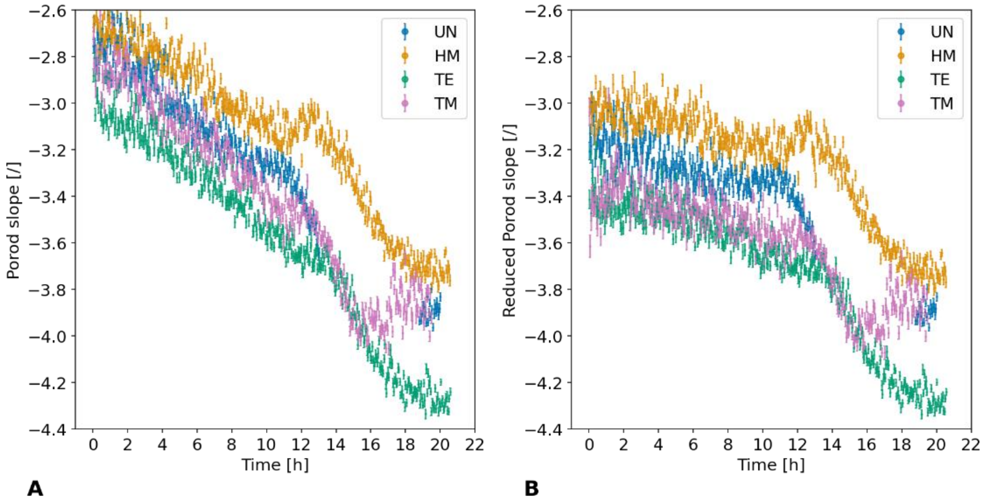

Fig. S5. The Porod slope of Pos. 2 of the unmodified UN (blue), hexamethyldisilazane-modified HM (orange), triethylchlorosilane-modified TE (green), and trimethylchlorosilane-modified TM (magenta) samples over the duration of the drying of calculated from the Q range of 0.31 Å^{-1} to 0.45 Å^{-1} for the measurements of Fig. S1 (**A**), and the hexane reduced data of Fig. S4 (**B**). The calculations are described in detail elsewhere.[1]

 This journal is © The Royal Society of Chemistry 2023

Note S3. Fractal slope evaluation

The tendencies of Fig. 3-E were visible in the fractal slope and reduced fractal slope values of Fig. S6-A and Fig. S6-B, which were described previously in Note S1. While the overall plot of the fractal dimension scattering model calculations is seemingly inversed to this calculation, this can be explained easily since instead of declining, negative slopes, the 'real' fractal dimension was evaluated. The overall development of the fractal dimension was very similar in comparison to the calculated fractal slopes, showing at first (I) the highest absolute values for the UN, followed by the TM, HM and TE samples. Other features, such as the wide curvature of the HM sample in region (III) in comparison to a relatively narrow course of the TM sample, were visible as well. On the contrary, the exact starting values of the samples differed slightly, with fractal slopes. Furthermore, while the UN sample showed a slight decrease in absolute fractal slope until the transition to a three-phase system (II), here the values were constant until the cutoff point. It is highly likely, that the scattering model calculations were more reliable, since the slope evaluations have had a higher error when the linearity of the X-ray scattering data was decreasing. This made an evaluation only possible if a linear decay was seen over a minimal Q range. Nonetheless, if a modeling of the X-ray scattering data is not feasible, the slope calculations in a double-logarithmic diagram seems to be a good approximation.

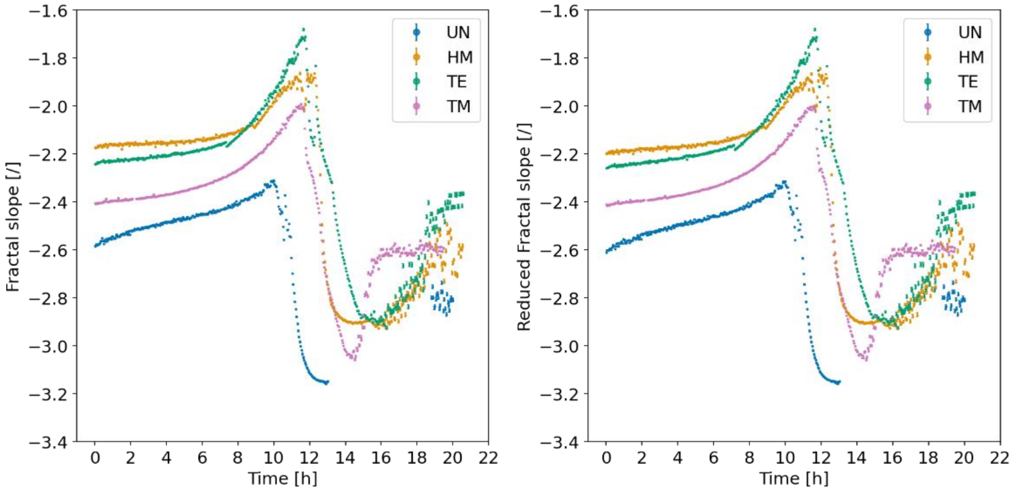

Fig. S6. The Fractal slope of Pos. 2 of the unmodified UN (blue), hexamethyldisilazane-modified HM (orange), triethylchlorosilane-modified TE (green), and trimethylchlorosilane-modified TM (magenta) samples over the duration of the drying of calculated from the Q range of 0.03 $Å^{-1}$ to 0.24 $Å^{-1}$ for the measurements of Fig. S1 (**A**), and the hexane reduced data of Fig. S4 (**B**). The calculations are described in detail elsewhere.[1]

Note S4. Supplementary results and discussions of scattering model parameters

Additional information was extracted from the scattering model and is shown in Fig. S7. The absolute values of the hexane scale are visualized in Fig. S7-B. While all samples started (I) at slightly different hexane scale values, they followed a similar trend, where an almost linear decay was visible until the cutoff point (II/III). Nonetheless, they differed quite heavily for the amount that was left after this linear decay, where the UN, HM, TE, and TM samples still had 0.19, 2e-07, 0.03, 0.16 of absolute hexane content at the cutoff point. Comparing this to the alternative calculation of Fig. S3, the trend was very similar, with a mostly linear decay. These similarities were not surprising, since the basic idea of calculating the hexane content from a Lorentzian fit in the WAXS region is identical, except for two principles. In the scattering model evaluation the contribution of two Lorentzian peaks for the (modified) silica backbone was assumed, and the position and width of the peaks were fitted to the last measurement, whereas the previous investigation (Fig. S3) chose one fixed peak. Additionally, these two peaks were directly constrained to the scaling factor of the fractal contribution and correspondingly to the volume fraction of the material, which should improve the fit of the hexane peak.

While the normalized SLD of the solvent (Fig. S7-D) was constrained to either pure hexane or the hexane peak, the SLD of the block was always kept constant. To this end, a previous study and its skeletal density evaluation was used to calculate a SLD of the block of $20.4\times 10^{-6}Å^{-2}$, $12.8\times 10^{-6}Å^{-2}$, and $13.6\times 10^{-6}Å^{-2}$ for the UN, both the HM and TE, as well as the TM samples, respectively. It has to be noted, that a high uncertainty was reported for these skeletal densities measured by helium pycnometry,[6] and a simplified molecular formula of SiO_2 was assumed for the samples. Assuming that the SLD of the block/material did not change throughout the experiment was another simplification. During the shrinkage and the drying of the samples, condensation reactions

of silanol end groups (SiOH) might lead to a slight increase of the skeletal density, which would increase the SLD of the block. Likewise, the condensation of silanol end groups to oxygen bridges produces water,[7] which would also increase the SLD of the solvent. The previous study showed that the TM sample had an unsignificant amount of silanol, whereas it was measurable for the HM, TE, and UN samples.[6] Since the condensation reactions would increase both the SLD of the solvent and the block, their difference would mostly stay the same. Additionally, a high contribution of water would be visible as a broad peak with a shoulder at around 3 $Å^{-1}$ in the WAXS region of the scattering measurements (Fig. S1).[8] Furthermore, it was shown in literature for polyimide aerogels that the SLD contrast could be affected by absorption of solvent by the material.[9] This could potentially decrease the contrast, but was assumed to be negligible. Nonetheless, the scattering contrast has an influence on the scale of the fractal contribution and could be a reason for the overestimation of the scale, which in turn constrained the intensity contribution of the (modified) silica, and subordinately the hexane contribution.

As mentioned, the contribution of the (modified) silica backbone was modeled by fitting two Lorentzian peaks to the WAXS region. Initially, the last measurement of the respective sample and sample position was used to fit the HWHM and position of the silica peaks, which are shown in Fig. S7-C, and Fig. S7-D. At this last point, it was assumed that the hexane contribution was negligible, and its peak did not overshadow the two silica peaks. The position of the first peak of the UN, HM, TE, and TM samples were determined to be 1.86 $Å^{-1}$, 1.22 $Å^{-1}$, 1.06 $Å^{-1}$, 1.20 $Å^{-1}$. Likewise, the 2nd peak was 1.61 $Å^{-1}$, 1.64 $Å^{-1}$, 1.64 $Å^{-1}$, and 1.66 $Å^{-1}$. The HWHM showed a high uncertainty. Afterwards, the HWHM and position of the two peaks were kept constant, and the scale of the peaks was constrained to the scaling factor of the fractal contribution. The latter describes the scaling factor of the spherical particles with fractal structure factor, which was proportional to the volume captured by the X-ray beam. Since a higher volume fraction refers to a less dense material where more matter is captured by the X-ray beam, the contribution of the (modified) silica backbone in the WAXS region had to be influenced as well. These results are shown in Fig. S7-C, and Fig. S7-D. At first (I/II) the hexane was completely overshadowing these silica contributions and they were therefore constrained to the scale of the fractal system. In other words, a shrinkage of the material is directly correlated to more silica being captured by the X-ray scattering.

The background parameter (Fig. S7-F) also slightly influenced the peaks in the WAXS region, where an increase in value would lead to a slight underestimation of the Lorentzian peak contributions. Nonetheless, this fit parameter was severely needed to improve the goodness of the fit, since a static background parameter would shift the full X-ray scattering profile to minimize the error. All samples started at around 0.09 mm cm^{-1} to 0.20 mm cm^{-1} (I) showed a slight decrease followed by an equal increase while shrinking, reaching roughly their starting values at the cutoff point (II/III). Following, another decrease was visible, which was more significant for the HM and TM sample, reaching roughly 0.04 mm cm^{-1} (IV). This large drop could be an indication of cracking of the material. On the other hand, the UN, and TE samples showed values of 0.08 mm cm^{-1} and 0.09 mm cm^{-1} (IV).

Immediately noticeable for Fig. S7-G were the differences in absolute values. While the UN sample showed values below 1, the HM and TE samples were in a range of 0.67 to 2.66 and 1.23 to 2.75. On the contrary, the TM sample was in a range of 2.88 to 9.05, which was severely higher. As mentioned before, the volume fraction of a previous study was fitted to the last measurement point,[6] which provides meaningful statements regarding the absolute scaling factor. Values higher than one indicate an underestimation of the volume fraction, vice versa an overestimation of the input porosity values. Since the porosity evaluations of the previous study have had a high uncertainty, the SAXS measurements could give another tendency about the validity. This could be an indication for the overestimation of the porosity values of the TM sample. The scaling factor of the fractal contribution, which was proportional to the volume fraction of the samples, indicated an overestimation of porosity values of another study. Nonetheless, the X-ray scattering measurements were not normalized to the width of the samples, and providing definite uncertainties was not possible. Therefore, this result was ambiguous because the data could not be normalized for the width of the sample.

Note S5. Goodness of the scattering model evaluation

The goodness of the fit can also be seen in Fig. S10, Fig. S11, Fig. S12, which show the scattering model data evaluated against the measurement data for Pos. 1, Pos. 2, Pos. 3, respectively, as well as Fig. S13 which shows the normalized residuals for these exemplary calculations. The normalized residuals were determined by subtracting the measurement data from the model and dividing this difference by the uncertainty of the measurement. As can be seen, besides some underestimation in the very low Q region, the measurement agreed with the scattering model evaluation. High residuals may only be seen for the WAXS region at the location of the crystalline peaks since they were generally not accounted for in the scattering model evaluation.

 This journal is © The Royal Society of Chemistry 2023

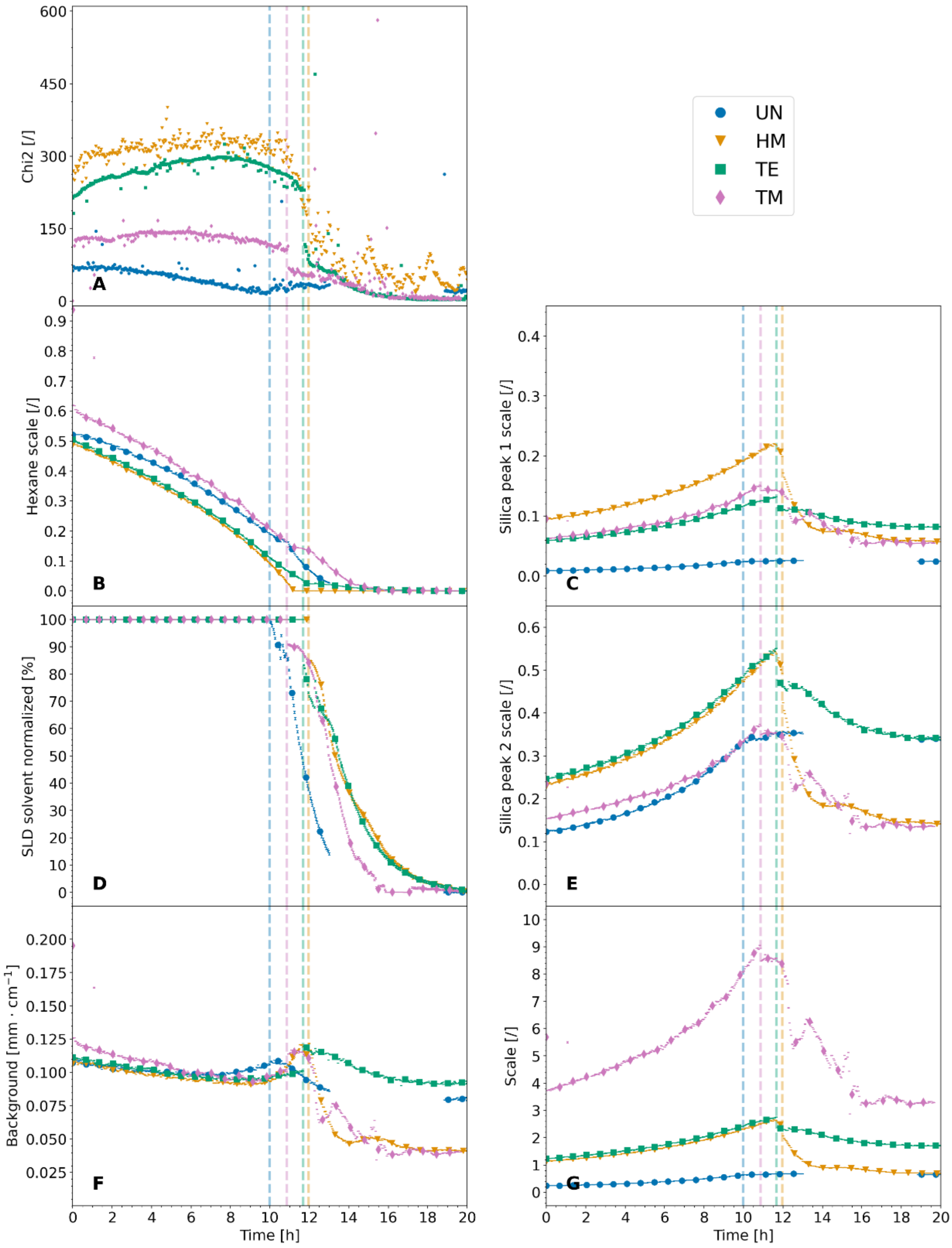

Fig. S7. The scattering model evaluation of the synchrotron X-ray scattering measurements of Pos. 2 of the unmodified UN (blue, circle), hexamethyldisilazane-modified HM (orange, triangle), triethylchlorosilane-modified TE (green, square), and trimethylchlorosilane-modified TM (magenta, diamond) samples with their corresponding standard deviation (bar line) are shown for the duration of the experiment. Additionally, dashed vertical lines in the color of the appropiate samples, show the estimated crossover of the two-phase system (silica-hexane) to a three-phase system (silica-hexane-air). The Chi-squared error (**A**) shows the goodness of the fit. The scale of the hexane peak (**B**) shows the evaporation. The scattering length density of the solvent (**D**) was constrained to the hexane peak in the three-phase system. The peaks of the (modified) silica backbone (**C, E**) were directly constrained to the scale/volume fraction of the samples. The background (**F**) and the scale of the fractal contribution (**G**) were left unrestricted.

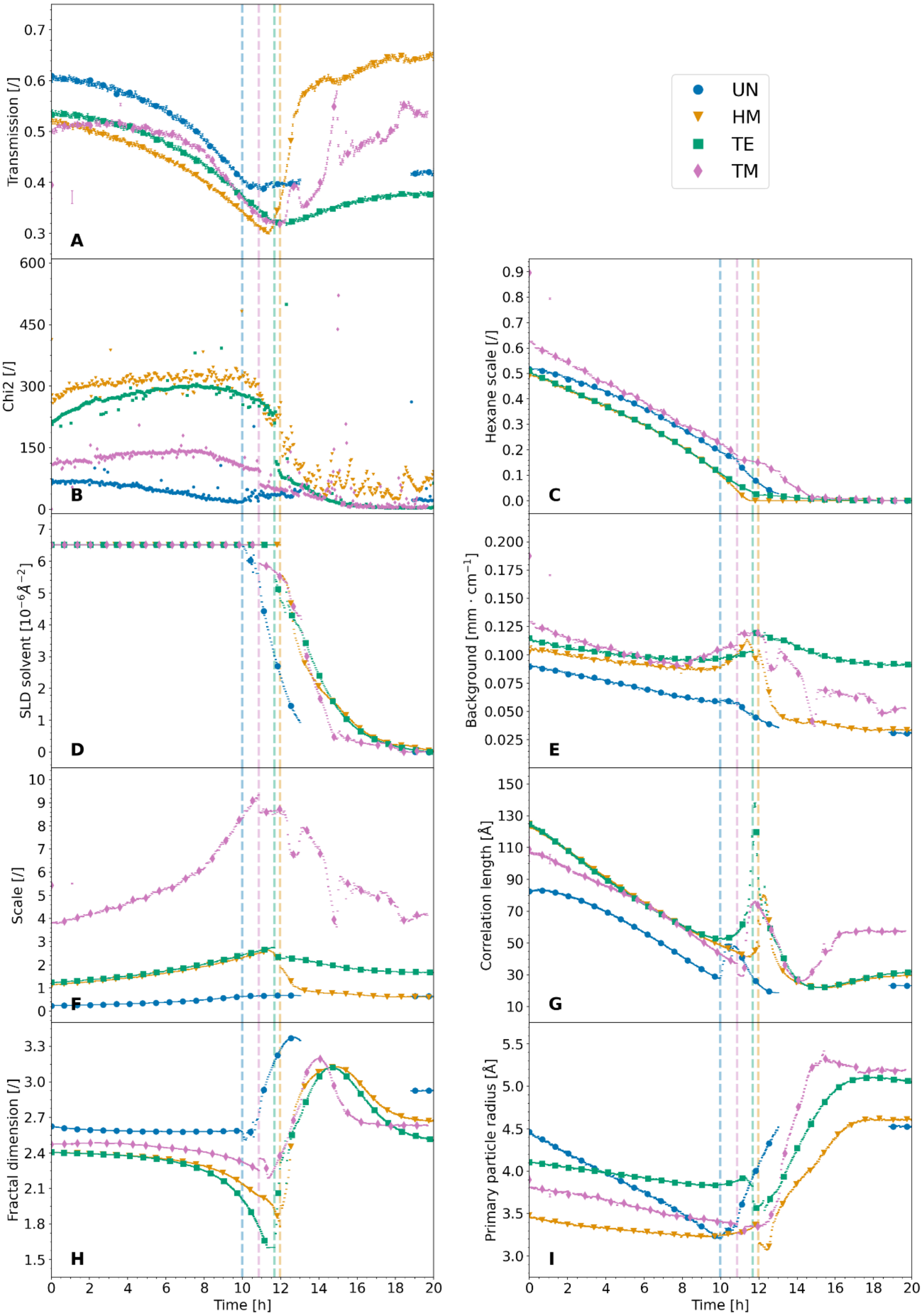

Fig. S8. The SasView evaluation of the Pos. 1 synchrotron X-ray scattering measurements of the unmodified UN (blue, circle), hexamethyldisilazane-modified HM (orange, triangle), triethylchlorosilane-modified TE (green, square), and trimethylchlorosilane-modified TM (magenta, diamond) samples with their corresponding standard deviation (bar line) are shown for the duration of the experiment, as well as the measured transmission of the samples (**A**). Additionally, dashed vertical lines in the color of the appropiate samples, show the estimated crossover of the two-phase system (silica-hexane) to a three-phase system (silica-hexane-air). The Chi-squared error (**B**) shows the goodness of the fit. The scale of the hexane peak (**C**) correlated with the scattering length density of the solvent (**D**), which was pure hexane at first, and was constrained to the hexane peak in the three-phase system. The background (**E**) and the scale of the fractal contribution (**F**) were left unrestricted. The correlation length (**G**), fractal dimension (**H**) and the primary particle radius (**I**) were evaluated from the fractal intensity contributions of the samples.

This journal is © The Royal Society of Chemistry 2023

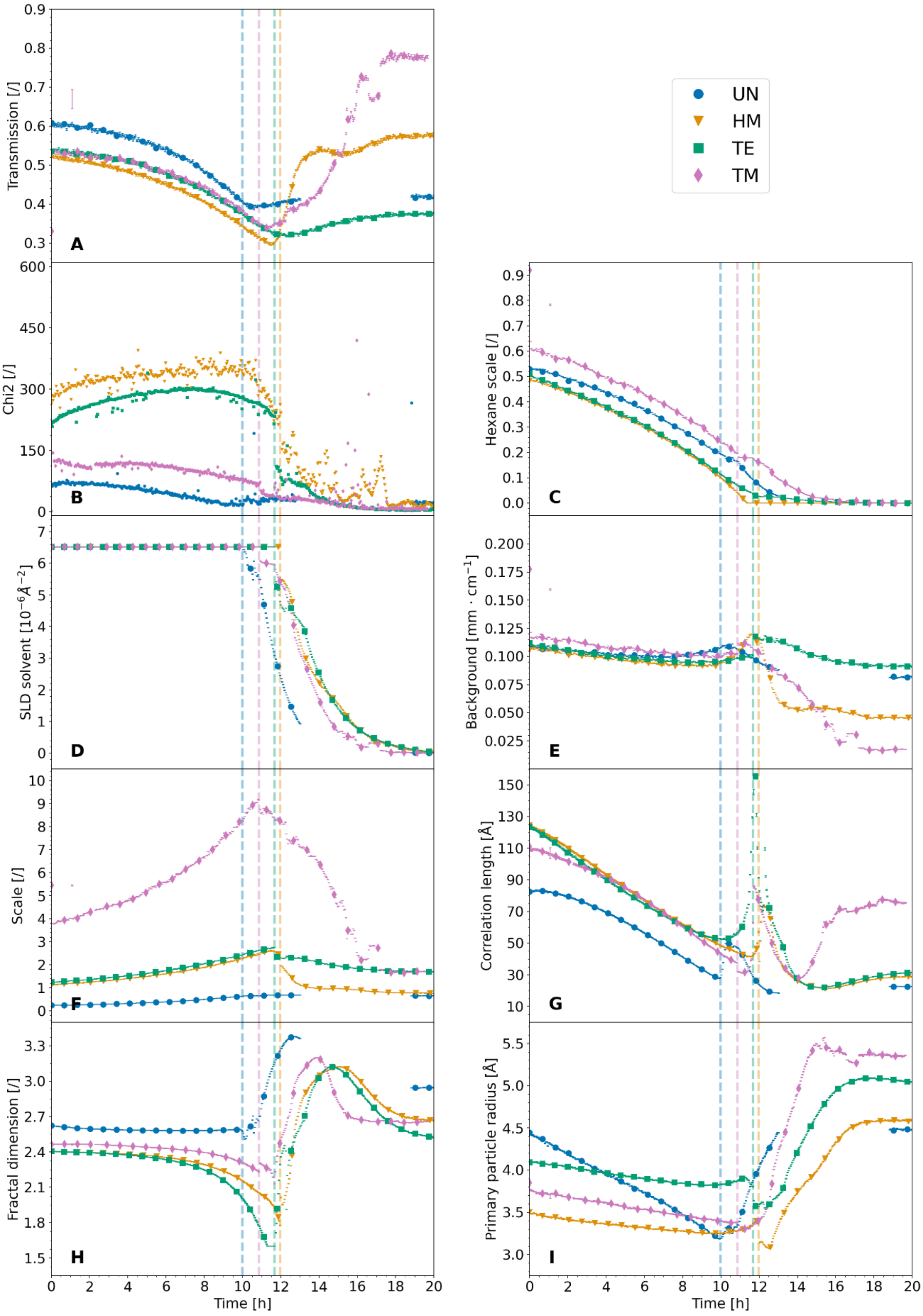

Fig. S9. The SasView evaluation of the Pos. 3 synchrotron X-ray scattering measurements of the unmodified UN (blue, circle), hexamethyldisilazane-modified HM (orange, triangle), triethylchlorosilane-modified TE (green, square), and trimethylchlorosilane-modified TM (magenta, diamond) samples with their corresponding standard deviation (bar line) are shown for the duration of the experiment, as well as the measured transmission of the samples (**A**). Additionally, dashed vertical lines in the color of the appropiate samples, show the estimated crossover of the two-phase system (silica-hexane) to a three-phase system (silica-hexane-air). The Chi-squared error (**B**) shows the goodness of the fit. The scale of the hexane peak (**C**) correlated with the scattering length density of the solvent (**D**), which was pure hexane at first, and was constrained to the hexane peak in the three-phase system. The background (**E**) and the scale of the fractal contribution (**F**) were left unrestricted. The correlation length (**G**), fractal dimension (**H**) and the primary particle radius (**I**) were evaluated from the fractal intensity contributions of the samples.

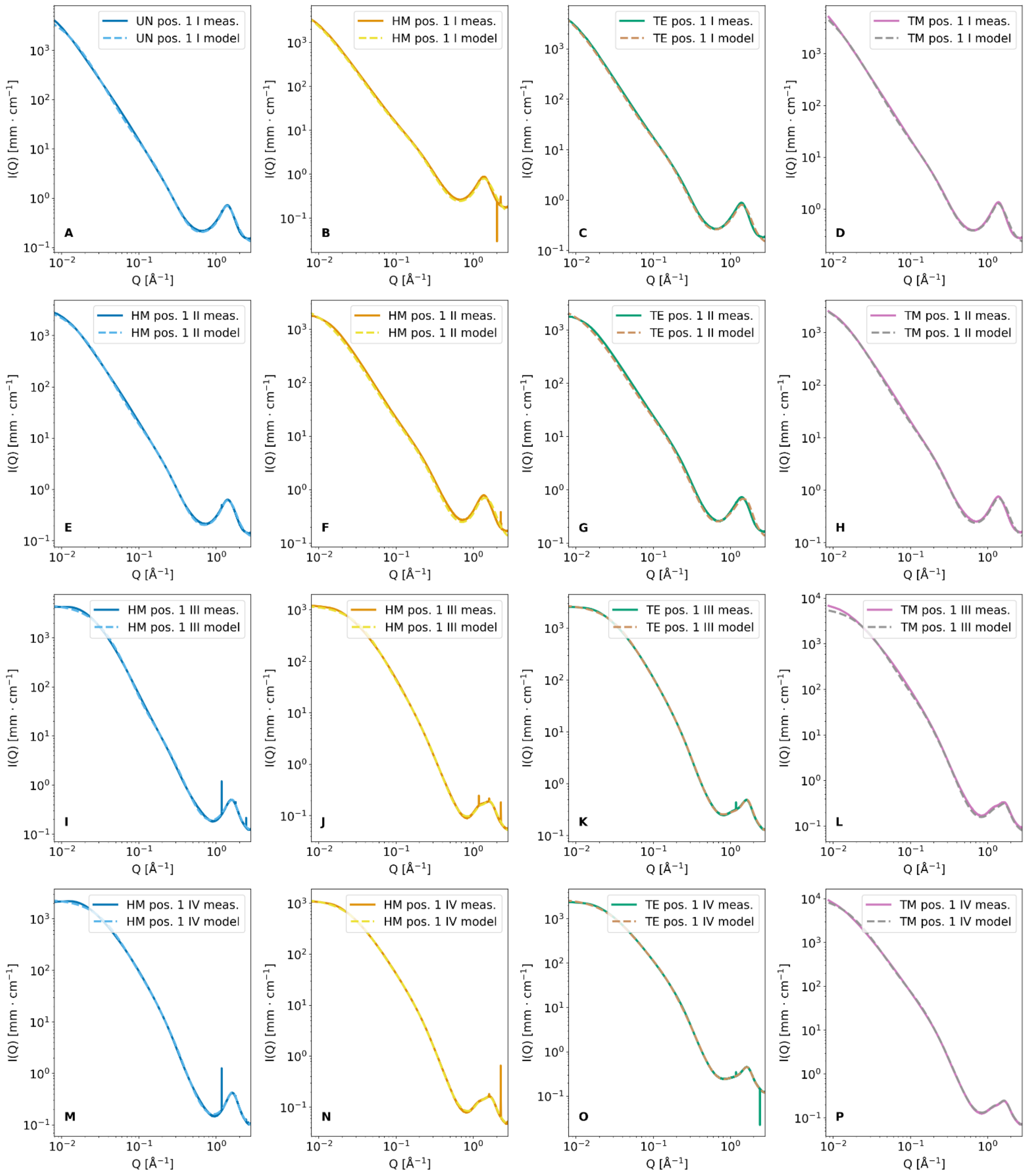

Fig. S10. The synchrotron X-ray scattering measurement dataset (straight line) compared to the SasView evaluation (dashed line) of Pos. 1 of **A-D)** the first measurement (I), **E-H)** one exemplary measurement of the two-phase system (II), **I-L)** one exemplary measurement of the three-phase system (III), **M-P)** the last measurement (IV). The goodness of the fit is shown for the unmodified UN (**A, E, I, M**), hexamethyldisilazane-modified HM (**B, F, J, N**), triethylchlorosilane-modified TE (**C, G, K, O**), and trimethylchlorosilane-modified TM (**D, H, L, P**) samples.

 This journal is © The Royal Society of Chemistry 2023

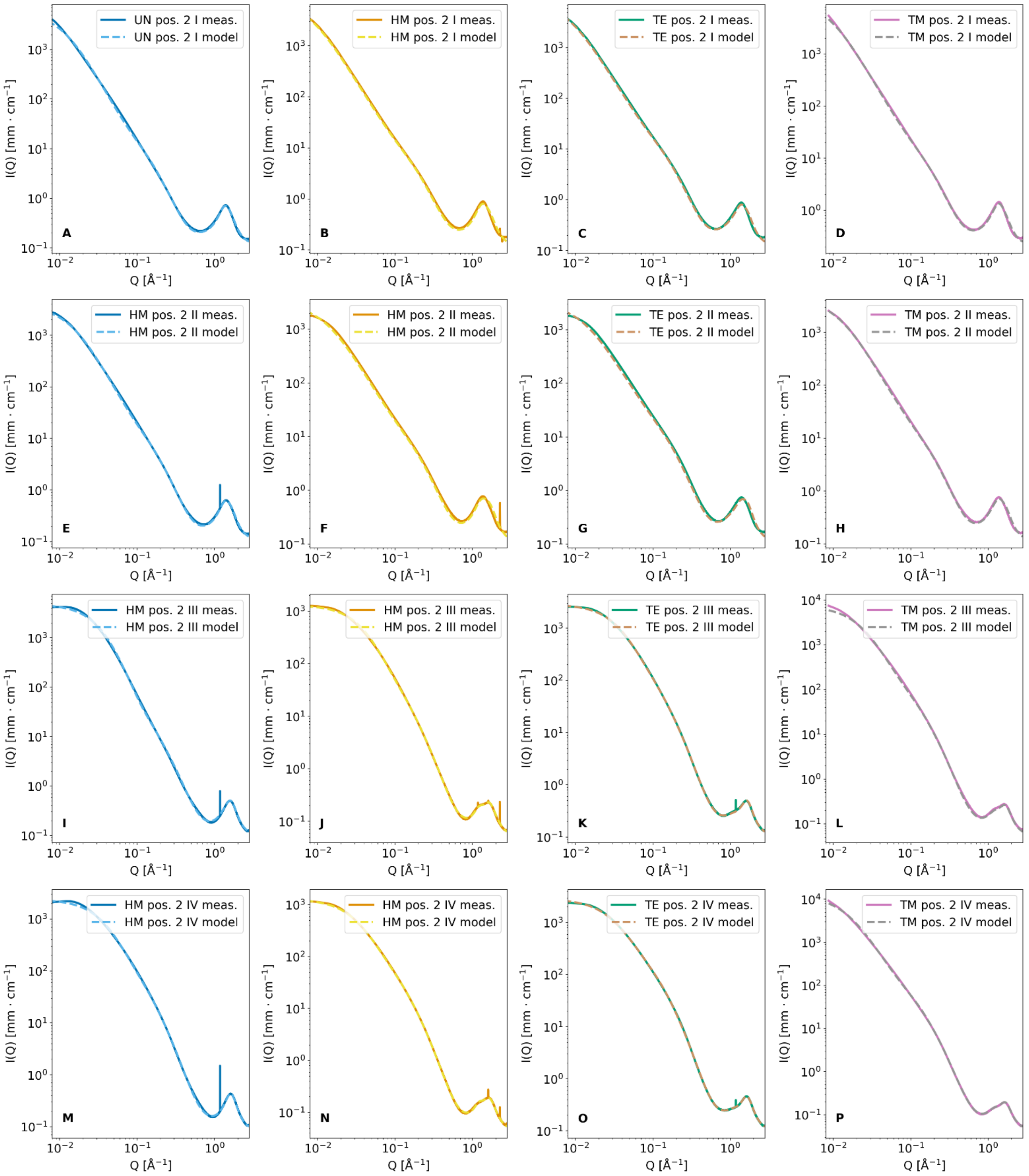

Fig. S11. The synchrotron X-ray scattering measurement dataset (straight line) compared to the SasView evaluation (dashed line) of Pos. 2 of **A-D)** the first measurement (I), **E-H)** one exemplary measurement of the two-phase system (II), **I-L)** one exemplary measurement of the three-phase system (III), **M-P)** the last measurement (IV). The goodness of the fit is shown for the unmodified UN (**A, E, I, M**), hexamethyldisilazane-modified HM (**B, F, J, N**), triethylchlorosilane-modified TE (**C, G, K, O**), and trimethylchlorosilane-modified TM (**D, H, L, P**) samples.

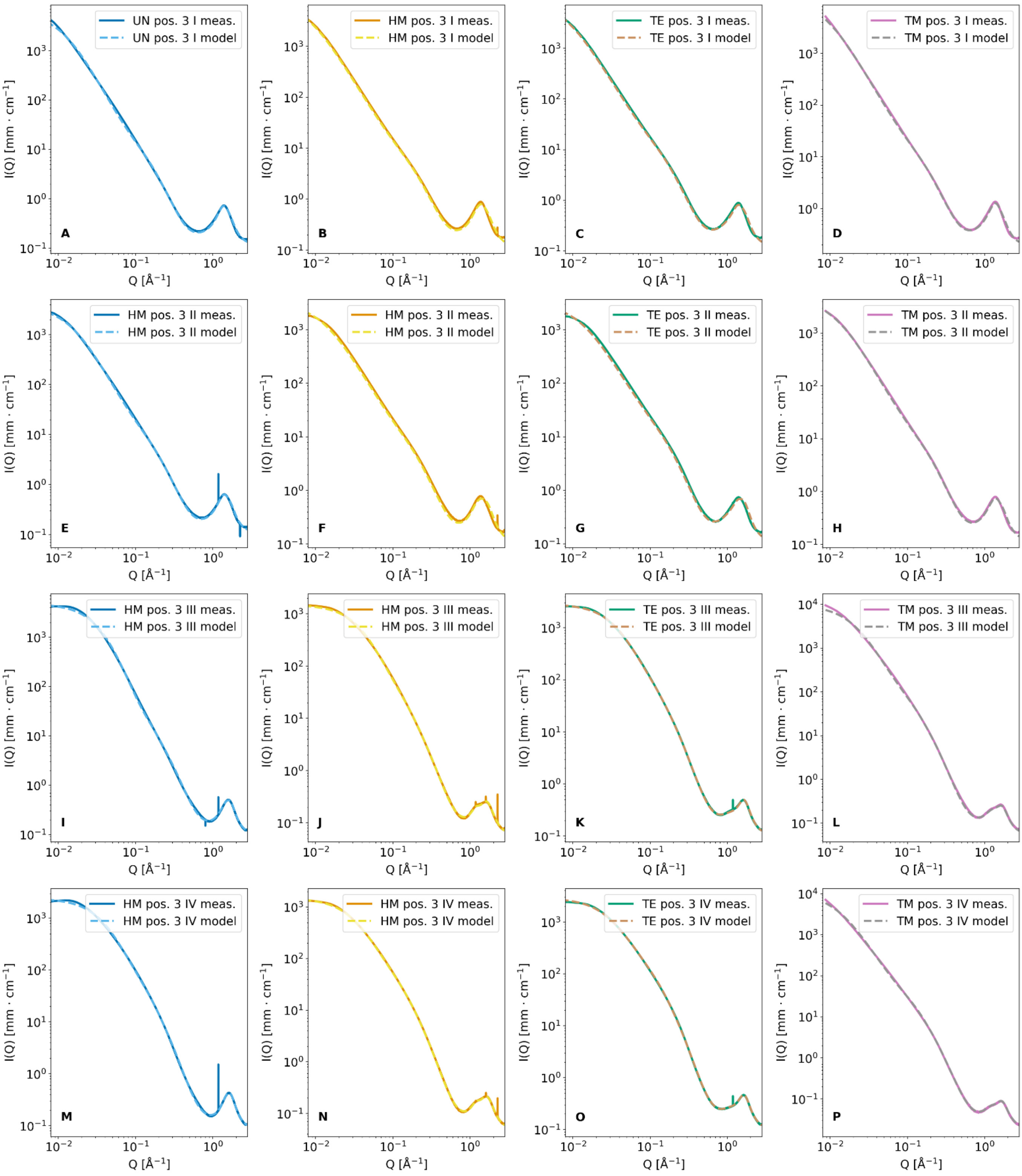

Fig. S12. The synchrotron X-ray scattering measurement dataset (straight line) compared to the SasView evaluation (dashed line) of Pos. 3 of **A-D)** the first measurement (I), **E-H)** one exemplary measurement of the two-phase system (II), **I-L)** one exemplary measurement of the three-phase system (III), **M-P)** the last measurement (IV). The goodness of the fit is shown for the unmodified UN (**A, E, I, M**), hexamethyldisilazane-modified HM (**B, F, J, N**), triethylchlorosilane-modified TE (**C, G, K, O**), and trimethylchlorosilane-modified TM (**D, H, L, P**) samples.

This journal is © The Royal Society of Chemistry 2023

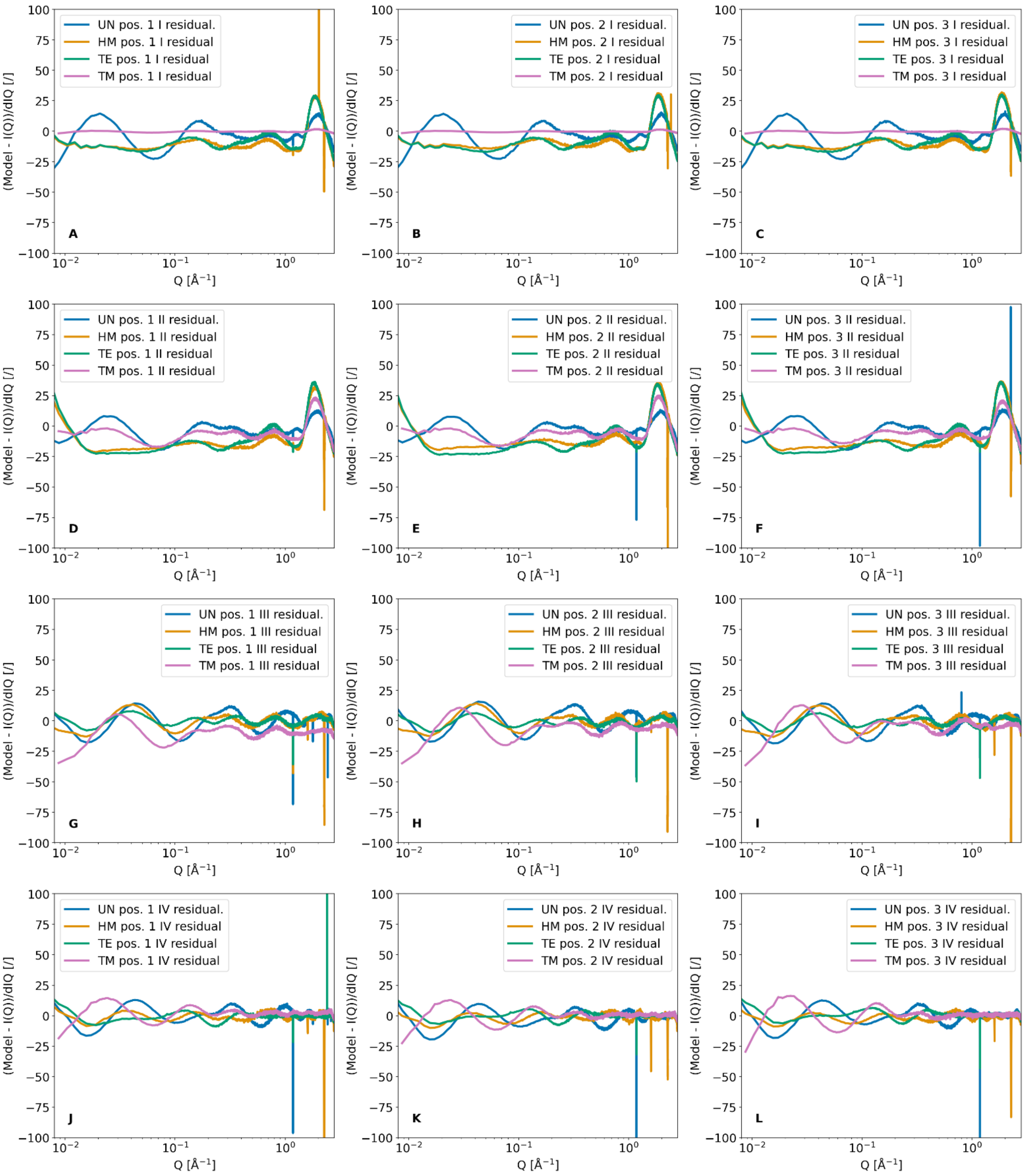

Fig. S13. The normalized residuals of the SasView evaluation calculated by the difference of the model and measurement data and divided by the error of the measurement of the unmodified UN (blue), hexamethyldisilazane-modified HM (orange), triethylchlorosilane-modified TE (green), and trimethylchlorosilane-modified TM (magenta) samples. The three positions were plotted for **A-C)** the first measurement (I), **D-F)** one exemplary measurement of the two-phase system (II), **G-I)** one exemplary measurement of the three-phase system (III), and **J-L)** the last measurement (IV).

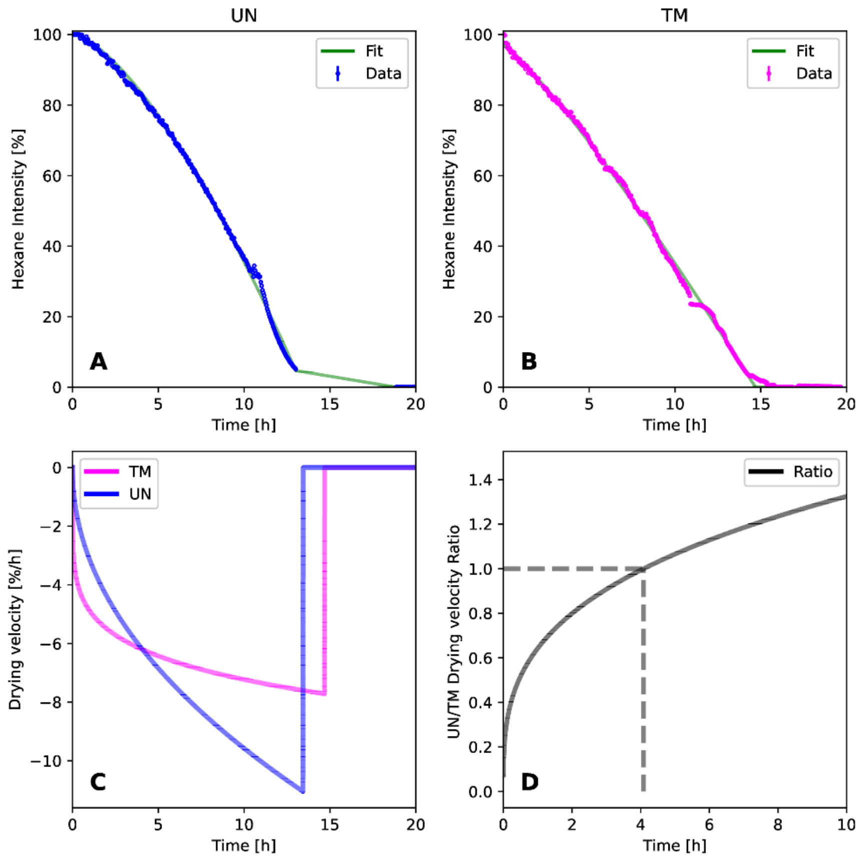

Fig. S14. Comparison of drying velocity for the UN (blue) and TM (magenta) samples. **A)** Time-dependant hexane intensity evolution (blue) and modelling (green) for the UN sample. **B)** Time-dependant hexane intensity evolution (magenta) and modelling (green) for the TM sample. **C)** Comparison between hexane content model derivatives for the UN (blue) and TM (magenta) samples. It can be observed that for the first 4 hours TM sample dries faster than UN. **D)** The UN/TM drying velocity ratio is less equal one for 4.08 h. Above this time, the drying velocity of the UN is faster than the TM sample, which is represented by values greater than one.

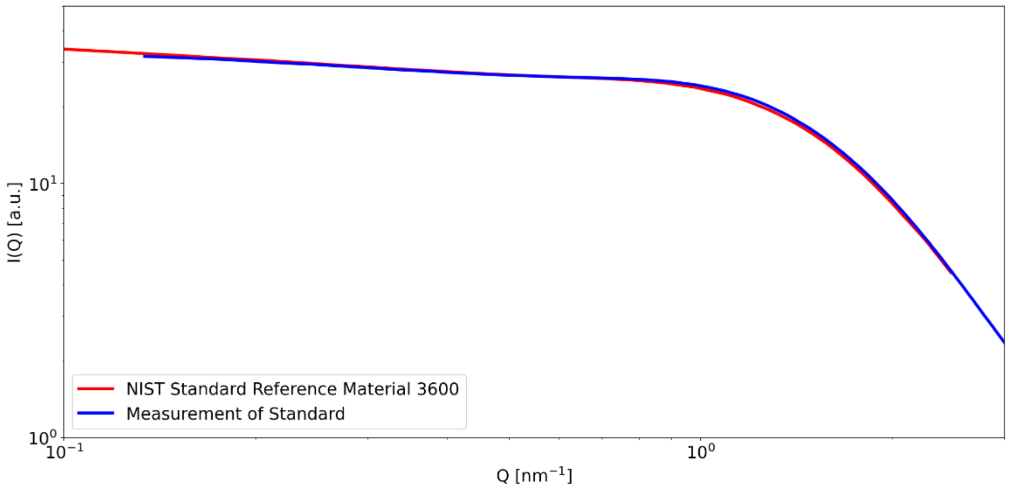

Fig. S15. Reported Standard reference material 3600 (red) of the National Institute of Standards and Technology (NIST) overlaid with a measurement of this standard (blue) for absolute intensity calibration.

 This journal is © The Royal Society of Chemistry 2023

Note S6. DOI of processed data

The processed (i.e., normalized) X-ray scattering data as well as the photographs captured throughout the experiments of the unmodified UN, hexamethyldisilazane-modified HM, triethylchlorosilane-modified TE, and trimethylchlorosilane-modified TM samples can be downloaded using the following link: https://doi.org/10.14279/depositonce-18443

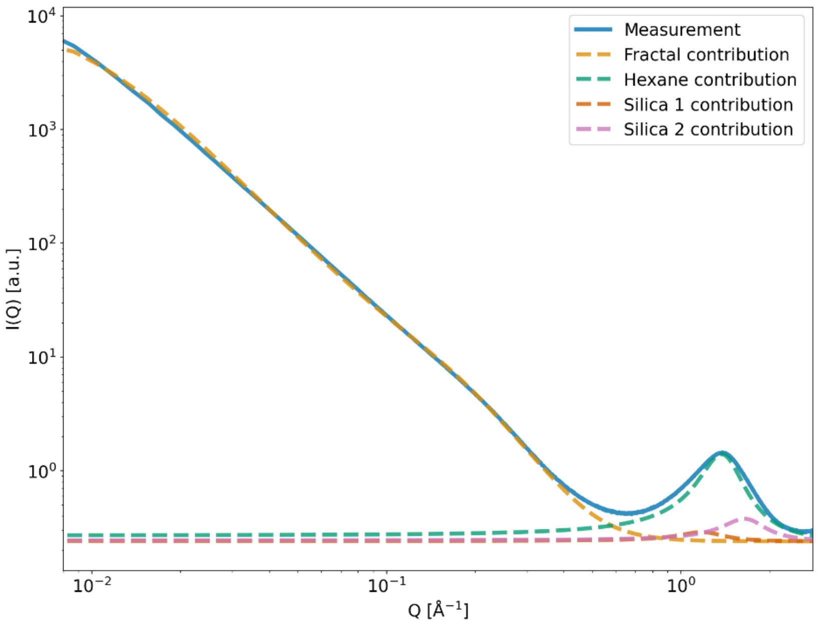

Fig. S16. Exemplary X-ray scattering measurement (blue) overlapped with the different intensity contributions, namely a fractal contribution (orange) and three Lorentzian peaks related to the hexane (green), as well as the silica backbone (red/magenta).

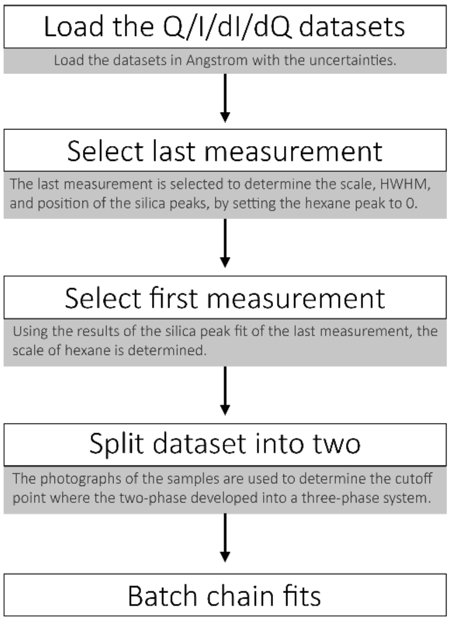

Fig. S17. Schematic of the procedure of the SasView evaluation: The measurement datasets are loaded, and the last measurement selected to determine the scale, half-width half-maximum (HWHM) of the silica peaks in the wide-angle X-ray scattering (WAXS) region. Afterwards, the first measurement is used to assess the initial hexane scale in the WAXS region. The cutoff point of the two-phase (silica-hexane) to the three-phase system (silica-hexane-air) was determined by evaluating the photographs. The two ranges of measurements were used for the batch evaluation.

Supporting information

Table S1. SasView evaluation input values of the last measurement points of the three positions for the unmodified UN, hexamethyldisilazane-modified HM, triethylchlorosilane-modified TE, and trimethylchlorosilane-modified TM samples. The fitting algorithm DREAM was used (--burn=100 --pop=10 --init=eps --thin=1 --steps=0), and a lognormal polydispersity was assumed for the radius of primary particles. (A_radius_pd.value = 0.5, A_radius_pd_n.value = 80, A_radius_pd_nsigma.value = 8). The background, scale of the fractal contribution, correlation length, radius, the two scales of the silica contributions, as well as their Half-width half-maximum (HWHM) and peak positions were fitted within the documented ranges.

		DREAM samples	scale	background			A_scale			A_volfraction	A_radius			A_radius polyd.	A_fractal_dim			A_cor_length			A_sld_block	A_sld_solvent	B_scale	C_scale			C_peak_pos			C_peak_hwhm			D_scale			D_peak_pos			D_peak_hwhm		
	Pos.	value	value	value	min.	max.	value	min.	max.	value	value	min.	max.	value	value	min.	max.	value	min.	max.	value	value	value	value	min.	max.	value	min.	max.	value	min.	max.	value	min.	max.	value	min.	max.	value	min.	max.
UN	1	1e+6	1	0.01	0	inf	1	0	inf	0.33	5	2	8	0.5	2.6	1.6	3.6	100	10	500	20.4	0	0	0.1	0	inf	1.2	0.9	2.85	0.3	0.1	inf	0.1	0	Inf	1.65	0.9	2.85	0.3	0.1	inf
	2	1e+6	1	0.01	0	inf	1	0	inf	0.33	5	2	8	0.5	2.6	1.6	3.6	100	10	500	20.4	0	0	0.1	0	inf	1.2	0.9	2.85	0.3	0.1	inf	0.1	0	Inf	1.65	0.9	2.85	0.3	0.1	inf
	3	1e+6	1	0.01	0	inf	1	0	inf	0.33	5	2	8	0.5	2.6	1.6	3.6	100	10	500	20.4	0	0	0.1	0	inf	1.2	0.9	2.85	0.3	0.1	2	0.1	0	Inf	1.65	0.9	2.85	0.3	0.1	2
HM	1	1e+6	1	0.01	0	inf	1	0	inf	0.41	5	2	8	0.5	2.6	1.6	3.6	100	10	500	12.8	0	0	0.1	0	inf	1.2	0.9	2.85	0.3	0.1	inf	0.1	0	Inf	1.65	0.9	2.85	0.3	0.1	inf
	2	1e+6	1	0.01	0	inf	1	0	inf	0.41	5	2	8	0.5	2.6	1.6	3.6	100	10	500	12.8	0	0	0.1	0	inf	1.2	0.9	2.85	0.3	0.1	inf	0.1	0	Inf	1.65	0.9	2.85	0.3	0.1	inf
	3	1e+6	1	0.01	0	inf	1	0	inf	0.41	5	2	8	0.5	2.6	1.6	3.6	100	10	500	12.8	0	0	0.1	0	inf	1.2	0.9	2.85	0.3	0.1	2	0.1	0	Inf	1.65	0.9	2.85	0.3	0.1	2
TE	1	1e+6	1	0.01	0	inf	1	0	inf	0.37	5	2	8	0.5	2.6	1.6	3.6	100	10	500	12.8	0	0	0.1	0	inf	1.2	0.9	2.85	0.3	0.1	inf	0.1	0	Inf	1.65	0.9	2.85	0.3	0.1	inf
	2	1e+6	1	0.01	0	inf	1	0	inf	0.37	5	2	8	0.5	2.6	1.6	3.6	100	10	500	12.8	0	0	0.1	0	inf	1.2	0.9	2.85	0.3	0.1	inf	0.1	0	Inf	1.65	0.9	2.85	0.3	0.1	inf
	3	1e+6	1	0.01	0	inf	1	0	inf	0.37	5	2	8	0.5	2.6	1.6	3.6	100	10	500	12.8	0	0	0.1	0	inf	1.2	0.9	2.85	0.3	0.1	2	0.1	0	Inf	1.65	0.9	2.85	0.3	0.1	2
TM	1	1e+6	1	0.01	0	inf	1	0	inf	0.09	5	2	8	0.5	2.6	1.6	3.6	100	10	500	13.6	0	0	0.1	0	inf	1.2	0.9	2.85	0.3	0.1	inf	0.1	0	Inf	1.65	0.9	2.85	0.3	0.1	inf
	2	1e+6	1	0.01	0	inf	1	0	inf	0.09	5	2	8	0.5	2.6	1.6	3.6	100	10	500	13.6	0	0	0.1	0	inf	1.2	0.9	2.85	0.3	0.1	inf	0.1	0	Inf	1.65	0.9	2.85	0.3	0.1	inf
	3	1e+6	1	0.01	0	inf	1	0	inf	0.09	5	2	8	0.5	2.6	1.6	3.6	100	10	500	13.6	0	0	0.1	0	inf	1.2	0.9	2.85	0.3	0.1	2	0.1	0	Inf	1.65	0.9	2.85	0.3	0.1	2

Table S2. SasView evaluation output values of the last measurement points of the three positions for the unmodified UN, hexamethyldisilazane-modified HM, triethylchlorosilane-modified TE, and trimethylchlorosilane-modified TM samples. The fitted values are shown with their respective 95 % confidence intervals, rounded to the 2nd digit (4th for the background). Additionally, the goodness of the fit is shown by the Chi-square (Chi2) values.

		Chi2	background		A_scale		A_radius		A_fractal_dim		A_cor_length		C_scale		C_peak_pos		C_peak_hwhm		D_scale		D_peak_pos		D_peak_hwhm	
	Pos.		value	P95 range	value	P95 range	value	P95 range	value	P95 range	value	P95 range	value	P95 range	value	P95 range	value	P95 range	value	P95 range	value	P95 range	value	P95 range
UN	1	23.0835	0.0305	0.0016/0.0754	0.63	0.63/0.64	4.54	4.49/4.58	2.92	2.86/2.95	23.26	22.52/24.71	0.05	0.01/0.35	2.12	0.99/2.77	1e+50	0.28/N.A.	0.35	0.01/0.35	1.62	1.03/2.72	0.30	0.30/N.A.
	2	22.4269	0.0799	0.0020/0.0815	0.65	0.63/0.65	4.50	4.46/4.65	2.94	2.74/2.96	22.67	22.14/28.00	0.02	0.01/0.35	1.86	1.00/2.69	0.27	0.24/N.A.	0.34	0.01/0.36	1.61	1.00/2.74	0.29	0.25/N.A.
	3	21.3348	0.0809	0.0410/0.0815	0.65	0.65/0.65	4.49	4.40/4.53	2.95	2.89/2.98	22.57	21.90/23.88	0.31	0.01/0.36	1.66	1.54/2.84	0.29	0.15/1.99	0.07	0.01/0.36	1.46	1.32/2.82	0.18	0.11/1.99
HM	1	101.0803	0.0325	0.0008/0.3197	0.58	0.57/0.58	4.59	4.49/4.69	2.69	2.60/2.72	29.10	28.25/32.33	0.04	0.01/0.16	1.20	1.20/2.85	0.20	0.17/4.14	0.12	0.01/0.15	1.62	1.14/2.85	0.39	0.13/N.A.
	2	19.1030	0.0392	0.0004/0.0364	0.67	0.66/0.68	4.57	4.48/4.73	2.68	2.52/2.74	28.39	26.81/34.24	0.06	0.01/0.18	1.22	1.04/2.84	0.21	0.22/N.A.	0.14	0.01/0.18	1.64	1.02/2.83	0.37	0.20/N.A.
	3	40.1067	0.0442	0.0020/0.0462	0.75	0.74/0.76	4.60	4.49/4.69	2.66	2.57/2.72	29.22	27.24/32.31	0.06	0.01/0.20	1.19	1.16/2.85	0.20	0.16/1.96	0.16	0.01/0.20	1.63	1.15/2.84	0.36	0.14/1.87
TE	1	46.9746	0.0895	0.0209/0.0905	1.68	1.65/1.69	5.02	4.99/5.18	2.51	2.39/2.57	32.03	29.83/36.44	0.34	0.11/0.34	1.64	1.09/1.66	0.32	0.24/1.34	0.08	0.08/0.31	1.05	0.98/1.66	0.38	0.23/1.73
	2	3.9786	0.0912	0.0057/0.0928	1.69	1.67/1.71	5.03	4.87/5.15	2.51	2.43/2.61	31.69	28.78/34.71	0.34	0.07/0.36	1.64	0.95/1.66	0.32	0.25/2.73	0.08	0.01/0.34	1.06	0.98/2.74	0.40	0.19/N.A.
	3	4.5146	0.0919	0.0544/0.0929	1.70	1.68/1.71	5.02	5.00/5.13	2.52	2.41/2.55	31.70	30.53/35.78	0.08	0.08/0.34	1.05	1.04/1.66	0.39	0.25/0.73	0.34	0.08/0.35	1.64	1.05/1.66	0.32	0.27/1.10
TM	1	4.1453	0.0526	0.0391/0.0544	4.14	4.11/4.15	5.20	5.18/5.26	2.63	2.60/2.65	57.56	55.66/62.00	0.06	0.05/0.18	1.20	1.19/1.72	0.27	0.20/0.52	0.17	0.05/0.18	1.65	1.18/1.72	0.33	0.21/0.52
	2	4.7015	0.0405	0.0031/0.0419	3.27	3.25/3.30	5.21	5.13/5.31	2.64	2.61/2.67	63.57	59.91/68.16	0.14	0.02/0.16	1.66	0.91/2.77	0.33	0.18/12.20	0.05	0.02/0.16	1.20	0.91/2.56	0.30	0.21/N.A.
	3	6.1418	0.0173	0.0145/0.0175	1.69	1.69/1.69	5.36	5.35/5.39	2.66	2.65/2.66	75.21	74.57/76.65	0.03	0.03/0.05	1.21	1.21/1.70	0.32	0.26/0.46	0.06	0.03/0.06	1.66	1.38/1.69	0.32	0.29/0.50

Table S3. SasView evaluation input values of the first measurement points of the three positions for the unmodified UN, hexamethyldisilazane-modified HM, triethylchlorosilane-modified TE, and trimethylchlorosilane-modified TM samples. The fitting algorithm DREAM was used (--burn=100 --pop=10 --init=eps --thin=1 --steps=0), and a lognormal polydispersity was assumed for the radius of primary particles. (A_radius_pd.value = 0.5, A_radius_pd_n.value = 80, A_radius_pd_nsigma.value = 8). The background, scale of the fractal contribution, correlation length, radius, the scale of the hexane contribution were fitted within the documented ranges.

		DREAM samples	scale	background			A_scale			A_volfraction	A_radius			A_radius polyd.	A_fractal_dim			A_cor_length			A_sld_block	A_sld_solvent	B_scale			B_peak_pos	B_peak_hwhm	C_scale	C_peak_pos	C_peak_hwhm	D_scale	D_peak_pos	D_peak_hwhm
	**Pos.**	**value**	**value**	**value**	**min.**	**max.**	**value**	**min.**	**max.**	**value**	**value**	**min.**	**max.**	**value**	**value**	**min.**	**max.**	**value**	**min.**	**max.**	**value**	**value**	**value**	**min.**	**max.**	**value**	**value**	**value**	**value**	**value**	**value**	**value**	**value**
**UN**	**1**	1e+6	1	0.0305	0	Inf	0.63	0	Inf	0.33	4.54	2	8	0.5	2.92	1.6	3.6	23.26	10	500	20.4	6.51	1	0	inf	1.3707	0.27434	$\frac{A_{scale} \cdot 0.0469928809483241}{0.63393411183473}$	2.12	1e+50	$\frac{A_{scale} \cdot 0.345483139062223}{0.63393411183473}$	1.62	0.30
	**2**	1e+6	1	0.0799	0	Inf	0.65	0	Inf	0.33	4.50	2	8	0.5	2.94	1.6	3.6	22.67	10	500	20.4	6.51	1	0	inf	1.3707	0.27434	$\frac{A_{scale} \cdot 0.0245224210317516}{0.64874229968752}$	1.86	0.27	$\frac{A_{scale} \cdot 0.340188096569123}{0.64874229968752}$	1.61	0.29
	**3**	1e+6	1	0.0809	0	Inf	0.65	0	Inf	0.33	4.49	2	8	0.5	2.95	1.6	3.6	22.57	10	500	20.4	6.51	1	0	inf	1.3707	0.27434	$\frac{A_{scale} \cdot 0.305706717686083}{0.650262941571917}$	1.66	0.29	$\frac{A_{scale} \cdot 0.0722308674879332}{0.650262941571917}$	1.46	0.18
**HM**	**1**	1e+6	1	0.0325	0	Inf	0.58	0	Inf	0.41	4.59	2	8	0.5	2.69	1.6	3.6	29.10	10	500	12.8	6.51	1	0	inf	1.3707	0.27434	$\frac{A_{scale} \cdot 0.0388075669119208}{0.579858815848481}$	1.20	0.20	$\frac{A_{scale} \cdot 0.122236963525714}{0.579858815848481}$	1.62	0.39
	**2**	1e+6	1	0.0392	0	Inf	0.67	0	Inf	0.41	4.57	2	8	0.5	2.68	1.6	3.6	28.39	10	500	12.8	6.51	1	0	inf	1.3707	0.27434	$\frac{A_{scale} \cdot 0.0559698910326038}{0.672774640497008}$	1.22	0.21	$\frac{A_{scale} \cdot 0.138308240411869}{0.672774640497008}$	1.64	0.37
	**3**	1e+6	1	0.0442	0	Inf	0.75	0	Inf	0.41	4.60	2	8	0.5	2.66	1.6	3.6	29.22	10	500	12.8	6.51	1	0	inf	1.3707	0.27434	$\frac{A_{scale} \cdot 0.0572698505976004}{0.75024565316198}$	1.19	0.20	$\frac{A_{scale} \cdot 0.160865723453778}{0.75024565316198}$	1.63	0.36
**TE**	**1**	1e+6	1	0.0895	0	Inf	1.68	0	Inf	0.37	5.02	2	8	0.5	2.51	1.6	3.6	32.03	10	500	12.8	6.51	1	0	inf	1.3707	0.27434	$\frac{A_{scale} \cdot 0.339386590051115}{1.67623384424382}$	1.64	0.32	$\frac{A_{scale} \cdot 0.0825401338821123}{1.67623384424382}$	1.05	0.38
	**2**	1e+6	1	0.0912	0	Inf	1.69	0	Inf	0.37	5.03	2	8	0.5	2.51	1.6	3.6	31.69	10	500	12.8	6.51	1	0	inf	1.3707	0.27434	$\frac{A_{scale} \cdot 0.337871115791165}{1.68719859604768}$	1.64	0.32	$\frac{A_{scale} \cdot 0.0810018100858287}{1.68719859604768}$	1.06	0.40
	**3**	1e+6	1	0.0919	0	Inf	1.70	0	Inf	0.37	5.02	2	8	0.5	2.52	1.6	3.6	31.70	10	500	12.8	6.51	1	0	inf	1.3707	0.27434	$\frac{A_{scale} \cdot 0.0793410447103211}{1.70000110300591}$	1.05	0.39	$\frac{A_{scale} \cdot 0.34267891183521}{1.70000110300591}$	1.64	0.32
**TM**	**1**	1e+6	1	0.0526	0	Inf	4.14	0	Inf	0.09	5.20	2	8	0.5	2.63	1.6	3.6	57.56	10	500	13.6	6.51	1	0	inf	1.3707	0.27434	$\frac{A_{scale} \cdot 0.0648646522431713}{4.13577393932143}$	1.20	0.27	$\frac{A_{scale} \cdot 0.171164269385688}{4.13577393932143}$	1.65	0.33
	**2**	1e+6	1	0.0405	0	Inf	3.27	0	Inf	0.09	5.21	2	8	0.5	2.64	1.6	3.6	63.57	10	500	13.6	6.51	1	0	inf	1.3707	0.27434	$\frac{A_{scale} \cdot 0.135091123359926}{3.27241593762015}$	1.66	0.33	$\frac{A_{scale} \cdot 0.0547691323415974}{3.27241593762015}$	1.20	0.30
	**3**	1e+6	1	0.0173	0	Inf	1.69	0	Inf	0.09	5.36	2	8	0.5	2.66	1.6	3.6	75.21	10	500	13.6	6.51	1	0	inf	1.3707	0.27434	$\frac{A_{scale} \cdot 0.0275830793660599}{1.68889187709127}$	1.21	0.32	$\frac{A_{scale} \cdot 0.0605536777006109}{1.68889187709127}$	1.66	0.32

Table S4. SasView evaluation output values of the first measurement points of the three positions for the unmodified UN, hexamethyldisilazane-modified HM, triethylchlorosilane-modified TE, and trimethylchlorosilane-modified TM samples. The fitted values are shown with their respective 95 % confidence intervals, rounded to the 2nd digit (4th for the background). Additionally, the goodness of the fit is shown by the Chi-square (Chi2) values.

			background		A_scale		A_radius		A_fractal_dim		A_cor_length		B_scale	
	**Pos.**	**Chi2**	**value**	**P95 range**	**value**	**P95 range**	**value**	**P95 range**	**value**	**P95 range**	**value**	**P95 range**	**value**	**P95 range**
**UN**	**1**	62.0259	0.0894	0.0892/0.0895	0.23	0.23/0.23	4.47	4.46/4.47	2.62	2.62/2.63	82.55	82.00/83.22	0.52	0.52/0.52
	**2**	63.1060	0.1074	0.1073/0.1075	0.24	0.24/0.24	4.46	4.45/4.47	2.62	2.62/2.62	82.67	82.06/83.29	0.52	0.52/0.52
	**3**	64.8841	0.1084	0.1083/0.1085	0.24	0.24/0.24	4.44	4.43/4.45	2.62	2.62/2.63	82.51	81.86/83.08	0.53	0.53/0.53
**HM**	**1**	413.5597	0.1024	0.1022/0.1025	1.10	1.10/1.10	3.48	3.48/3.49	2.40	2.40/2.40	125.10	124.10/126.27	0.49	0.49/0.49
	**2**	252.3404	0.1066	0.1064/0.1067	1.12	1.11/1.12	3.47	3.47/3.48	2.41	2.40/2.41	124.07	122.99/125.11	0.49	0.49/0.49
	**3**	263.5055	0.1057	0.1055/0.1058	1.11	1.11/1.11	3.50	3.50/3.51	2.40	2.40/2.41	124.24	123.27/125.39	0.48	0.48/0.48
**TE**	**1**	208.1222	0.1147	0.1145/0.1148	1.24	1.24/1.24	4.10	4.10/4.11	2.41	2.40/2.41	124.66	123.33/125.54	0.51	0.51/0.51
	**2**	213.0286	0.1114	0.1112/0.1115	1.23	1.23/1.23	4.10	4.10/4.11	2.40	2.40/2.41	124.18	123.05/125.24	0.50	0.50/0.51
	**3**	215.5137	0.1119	0.1118/0.1120	1.25	1.25/1.25	4.09	4.09/4.10	2.40	2.40/2.41	123.32	122.33/124.50	0.51	0.51/0.51
**TM**	**1**	0.6928	0.1872	0.1849/0.1901	5.43	5.36/5.50	3.90	3.82/3.98	2.47	2.44/2.50	108.90	96.73/125.80	0.90	0.88/0.91
	**2**	0.5338	0.1952	0.1915/0.1981	5.69	5.60/5.78	3.84	3.74/3.94	2.47	2.44/2.50	107.29	94.22/130.15	0.94	0.92/0.96
	**3**	1.0650	0.1775	0.1753/0.1796	5.45	5.39/5.51	3.85	3.79/3.92	2.46	2.44/2.49	111.01	99.95/125.52	0.92	0.91/0.92

This journal is © The Royal Society of Chemistry 2023

Table S5. SasView evaluation input values of the first batch measurements of the three positions for the unmodified UN, hexamethyldisilazane-modified HM, triethylchlorosilane-modified TE, and trimethylchlorosilane-modified TM samples. The fitting algorithm DREAM was used (--burn=100 --pop=10 --init=eps --thin=1 --steps=0), and a lognormal polydispersity was assumed for the radius of primary particles. (A_radius_pd.value = 0.5, A_radius_pd_n.value = 80, A_radius_pd_nsigma.value = 8). The background, scale of the fractal contribution, correlation length, radius, and the scale of the hexane contribution were fitted within the documented ranges. A chain batch fit and therefore the previous measurement outputs were used.

		DREAM samples	scale	background			A_scale			A_volfraction	A_radius			A_radius polyd.	A_fractal_dim			A_cor_length			A_sld_block	A_sld_solvent	B_scale			B_peak_pos	B_peak_hwhm	C_scale	C_peak_pos	C_peak_hwhm	D_scale	D_peak_pos	D_peak_hwhm
	Pos.	value	value	value	min.	max.	value	min.	max.	value	value	min.	max.	value	value	min.	max.	value	min.	max.	value	value	value	min.	max.	value	value	value	value	value	value	value	value
UN	1	1e+5	1	0.0894	0	Inf	0.23	0	Inf	0.33	4.47	2	8	0.5	2.62	1.6	3.6	82.55	10	500	20.4	6.51	0.52	0	0.52	1.3707	0.27434	$\frac{A_{scale} \cdot 0.0469928809483241}{0.63393411183473}$	2.12	1e+50	$\frac{A_{scale} \cdot 0.345483139062223}{0.63393411183473}$	1.62	0.30
	2	1e+5	1	0.1074	0	Inf	0.24	0	Inf	0.33	4.46	2	8	0.5	2.62	1.6	3.6	82.67	10	500	20.4	6.51	0.52	0	0.52	1.3707	0.27434	$\frac{A_{scale} \cdot 0.0245224210317516}{0.64874229968752}$	1.86	0.27	$\frac{A_{scale} \cdot 0.340188096569123}{0.64874229968752}$	1.61	0.29
	3	1e+5	1	0.1084	0	Inf	0.24	0	Inf	0.33	4.44	2	8	0.5	2.62	1.6	3.6	82.51	10	500	20.4	6.51	0.53	0		1.3707	0.27434	$\frac{A_{scale} \cdot 0.305706717686083}{0.650262941571917}$	1.66	0.29	$\frac{A_{scale} \cdot 0.0722308674879332}{0.650262941571917}$	1.46	0.18
HM	1	1e+5	1	0.1024	0	Inf	1.10	0	Inf	0.41	3.48	2	8	0.5	2.40	1.6	3.6	125.10	10	500	12.8	6.51	0.49	0	0.49	1.3707	0.27434	$\frac{A_{scale} \cdot 0.0388075669119208}{0.579858815848481}$	1.20	0.20	$\frac{A_{scale} \cdot 0.122236963525714}{0.579858815848481}$	1.62	0.39
	2	1e+5	1	0.1066	0	Inf	1.12	0	Inf	0.41	3.47	2	8	0.5	2.41	1.6	3.6	124.07	10	500	12.8	6.51	0.49	0	0.49	1.3707	0.27434	$\frac{A_{scale} \cdot 0.0559698910326038}{0.672774640497008}$	1.22	0.21	$\frac{A_{scale} \cdot 0.138308240411869}{0.672774640497008}$	1.64	0.37
	3	1e+5	1	0.1057	0	Inf	1.11	0	Inf	0.41	3.50	2	8	0.5	2.40	1.6	3.6	124.24	10	500	12.8	6.51	0.48	0		1.3707	0.27434	$\frac{A_{scale} \cdot 0.0572698505976004}{0.75024565316198}$	1.19	0.20	$\frac{A_{scale} \cdot 0.160865723453778}{0.75024565316198}$	1.63	0.36
TE	1	1e+5	1	0.1147	0	Inf	1.24	0	Inf	0.37	4.10	2	8	0.5	2.41	1.6	3.6	124.66	10	500	12.8	6.51	0.51	0	0.51	1.3707	0.27434	$\frac{A_{scale} \cdot 0.339386590051115}{1.67623384424382}$	1.64	0.32	$\frac{A_{scale} \cdot 0.0825401338821123}{1.67623384424382}$	1.05	0.38
	2	1e+5	1	0.1114	0	Inf	1.23	0	Inf	0.37	4.10	2	8	0.5	2.40	1.6	3.6	124.18	10	500	12.8	6.51	0.50	0	0.50	1.3707	0.27434	$\frac{A_{scale} \cdot 0.337871115791165}{1.68719859604768}$	1.64	0.32	$\frac{A_{scale} \cdot 0.0810018100858287}{1.68719859604768}$	1.06	0.40
	3	1e+5	1	0.1119	0	Inf	1.25	0	Inf	0.37	4.09	2	8	0.5	2.40	1.6	3.6	123.32	10	500	12.8	6.51	0.51	0		1.3707	0.27434	$\frac{A_{scale} \cdot 0.0793410447103211}{1.70000110300591}$	1.05	0.39	$\frac{A_{scale} \cdot 0.34267891183521}{1.70000110300591}$	1.64	0.32
TM	1	1e+5	1	0.1872	0	Inf	5.43	0	Inf	0.09	3.90	2	8	0.5	2.47	1.6	3.6	108.90	10	500	13.6	6.51	0.90	0	0.90	1.3707	0.27434	$\frac{A_{scale} \cdot 0.0648646522431713}{4.13577393932143}$	1.20	0.27	$\frac{A_{scale} \cdot 0.171164269385688}{4.13577393932143}$	1.65	0.33
	2	1e+5	1	0.1952	0	Inf	5.69	0	Inf	0.09	3.84	2	8	0.5	2.47	1.6	3.6	107.29	10	500	13.6	6.51	0.94	0	0.94	1.3707	0.27434	$\frac{A_{scale} \cdot 0.135091123359926}{3.27241593762015}$	1.66	0.33	$\frac{A_{scale} \cdot 0.0547691323415974}{3.27241593762015}$	1.20	0.30
	3	1e+5	1	0.1775	0	Inf	5.45	0	Inf	0.09	3.85	2	8	0.5	2.46	1.6	3.6	111.01	10	500	13.6	6.51	0.92	0		1.3707	0.27434	$\frac{A_{scale} \cdot 0.0275830793660599}{1.68889187709127}$	1.21	0.32	$\frac{A_{scale} \cdot 0.0605536777006109}{1.68889187709127}$	1.66	0.32

Table S6. SasView evaluation input values of the first batch measurements of the three positions for the unmodified UN, hexamethyldisilazane-modified HM, triethylchlorosilane-modified TE, and trimethylchlorosilane-modified TM samples. The fitting algorithm DREAM was used (--burn=100 --pop=10 --init=eps --thin=1 --steps=0), and a lognormal polydispersity was assumed for the radius of primary particles. (A_radius_pd.value = 0.5, A_radius_pd_n.value = 80, A_radius_pd_nsigma.value = 8). The background, scale of the fractal contribution, correlation length, radius, the scale of the hexane contribution were fitted within the documented ranges. A chain batch fit and therefore the previous measurement outputs were used. The values of the B_scale were cut off after the 2nd digit, although the full number was used for the input.

		DREAM samples	scale	background			A_scale			A_volfraction	A_radius			A_radius polyd.	A_fractal_dim			A_cor_length			A_sld_block	A_sld_solvent	B_scale			B_peak_pos	B_peak_hwhm	C_scale	C_peak_pos	C_peak_hwhm	D_scale	D_peak_pos	D_peak_hwhm
	Pos.	value	value	value	min.	max.	value	min.	max.	value	value	min.	max.	value	value	min.	max.	value	min.	max.	value	value	value	min.	max.	value	value	value	value	value	value	value	value
UN	1	1e+5	1	0.0579	0	Inf	0.63	0	Inf	0.33	3.22	2	8	0.5	2.57	1.6	3.6	28.29	10	500	20.4	$\frac{B_{scale} \cdot 6.51}{0.191236794241103}$	0.19..	0	0.19..	1.3707	0.27434	$\frac{A_{scale} \cdot 0.0469928809483241}{0.63393411183473}$	2.12	1e+50	$\frac{A_{scale} \cdot 0.345483139062223}{0.63393411183473}$	1.62	0.30
	2	1e+5	1	0.1057	0	Inf	0.63	0	Inf	0.33	3.20	2	8	0.5	2.57	1.6	3.6	28.48	10	500	20.4	$\frac{B_{scale} \cdot 6.51}{0.190539184071713}$	0.19..	0	0.19..	1.3707	0.27434	$\frac{A_{scale} \cdot 0.0245224210317516}{0.64874229968752}$	1.86	0.27	$\frac{A_{scale} \cdot 0.340188096569123}{0.64874229968752}$	1.61	0.29
	3	1e+5	1	0.1074	0	Inf	0.64	0	Inf	0.33	3.19	2	8	0.5	2.57	1.6	3.6	28.45	10	500	20.4	$\frac{B_{scale} \cdot 6.51}{0.193521670240227}$	0.19..	0	0.19..	1.3707	0.27434	$\frac{A_{scale} \cdot 0.305706717686083}{0.650262941571917}$	1.66	0.29	$\frac{A_{scale} \cdot 0.0722308674879332}{0.650262941571917}$	1.46	0.18
HM	1	1e+5	1	0.0921	0	Inf	2.21	0	Inf	0.41	3.39	2	8	0.5	1.78	1.6	3.6	63.94	10	500	12.8	$\frac{B_{scale} \cdot 6.51}{6.43020896614489e-07}$	6.43e-7	0	6.43e-7	1.3707	0.27434	$\frac{A_{scale} \cdot 0.0388075669119208}{0.579858815848481}$	1.20	0.20	$\frac{A_{scale} \cdot 0.122236963525714}{0.579858815848481}$	1.62	0.39
	2	1e+5	1	0.1089	0	Inf	2.40	0	Inf	0.41	3.41	2	8	0.5	1.76	1.6	3.6	58.54	10	500	12.8	$\frac{B_{scale} \cdot 6.51}{2.26777032709011e-07}$	2.26e-7	0	2.26e-7	1.3707	0.27434	$\frac{A_{scale} \cdot 0.0559698910326038}{0.672774640497008}$	1.22	0.21	$\frac{A_{scale} \cdot 0.138308240411869}{0.672774640497008}$	1.64	0.37
	3	1e+5	1	0.1047	0	Inf	2.31	0	Inf	0.41	3.43	2	8	0.5	1.78	1.6	3.6	57.07	10	500	12.8	$\frac{B_{scale} \cdot 6.51}{6.0071738180524e-07}$	6.01e-7	0	6.01e-7	1.3707	0.27434	$\frac{A_{scale} \cdot 0.0572698505976004}{0.75024565316198}$	1.19	0.20	$\frac{A_{scale} \cdot 0.160865723453778}{0.75024565316198}$	1.63	0.36
TE	1	1e+5	1	0.1022	0	Inf	2.73	0	Inf	0.37	3.82	2	8	0.5	1.60	1.6	3.6	167.43	10	500	12.8	$\frac{B_{scale} \cdot 6.51}{0.0316040112792858}$	0.03..	0	0.03..	1.3707	0.27434	$\frac{A_{scale} \cdot 0.339386590051115}{1.67623384424382}$	1.64	0.32	$\frac{A_{scale} \cdot 0.0825401338821123}{1.67623384424382}$	1.05	0.38
	2	1e+5	1	0.1010	0	Inf	2.75	0	Inf	0.37	3.82	2	8	0.5	1.60	1.6	3.6	307.30	10	500	12.8	$\frac{B_{scale} \cdot 6.51}{0.0339611180037783}$	0.03..	0	0.03..	1.3707	0.27434	$\frac{A_{scale} \cdot 0.337871115791165}{1.68719859604768}$	1.64	0.32	$\frac{A_{scale} \cdot 0.0810018100858287}{1.68719859604768}$	1.06	0.40
	3	1e+5	1	0.1005	0	Inf	2.75	0	Inf	0.37	3.81	2	8	0.5	1.6	1.6	3.6	199.88	10	500	12.8	$\frac{B_{scale} \cdot 6.51}{0.0361998789978834}$	0.03..	0	0.03..	1.3707	0.27434	$\frac{A_{scale} \cdot 0.0793410447103211}{1.70000110300591}$	1.05	0.39	$\frac{A_{scale} \cdot 0.34267891183521}{1.70000110300591}$	1.64	0.32
TM	1	1e+5	1	0.1083	0	Inf	9.35	0	Inf	0.09	3.37	2	8	0.5	2.25	1.6	3.6	37.00	10	500	13.6	$\frac{B_{scale} \cdot 6.51}{0.177951429715859}$	0.18..	0	0.18..	1.3707	0.27434	$\frac{A_{scale} \cdot 0.0648646522431713}{4.13577393932143}$	1.20	0.27	$\frac{A_{scale} \cdot 0.171164269385688}{4.13577393932143}$	1.65	0.33
	2	1e+5	1	0.1006	0	Inf	8.94	0	Inf	0.09	3.41	2	8	0.5	2.23	1.6	3.6	38.17	10	500	13.6	$\frac{B_{scale} \cdot 6.51}{0.160705576788826}$	0.16..	0	0.16..	1.3707	0.27434	$\frac{A_{scale} \cdot 0.135091123359926}{3.27241593762015}$	1.66	0.33	$\frac{A_{scale} \cdot 0.0547691323415974}{3.27241593762015}$	1.20	0.30
	3	1e+5	1	0.1022	0	Inf	9.16	0	Inf	0.09	3.38	2	8	0.5	2.23	1.6	3.6	37.45	10	500	13.6	$\frac{B_{scale} \cdot 6.51}{0.194725292339111}$	0.19..	0	0.19..	1.3707	0.27434	$\frac{A_{scale} \cdot 0.0275830793660599}{1.68889187709127}$	1.21	0.32	$\frac{A_{scale} \cdot 0.0605536777006109}{1.68889187709127}$	1.66	0.32

 This journal is © The Royal Society of Chemistry 2023

Note S7. Creating a custom SasView model

With SasView's model editor, a "fractal" model "A", was combined with three "peak_lorentz" contributions, with "B", "C", and "D" being three individual peaks in the wide-angle X-ray scattering (WAXS) region. While "B" was a contribution of hexane, "C" and "D" originated from the silica structure. The python library 'sasmodels' was used for the evaluation. The combined model inherited a scaling factor ("scale") and a background ("background"). The "fractal" contribution $\mathrm{I}_{\mathrm{fractal}}$ was composed of a scale ("A_scale"), volume fraction of the silica backbone and the solvent ("A_volfraction"), radius of primary particles ("A_radius") with a lognormal polydispersity ("A_PD_ratio"), Fractal dimension ("A_fractal_dim"), and size of cluster also known as correlation length ("A_cor_length"). Furthermore, a scattering length density (SLD) contrast was a combination of the silica block ("A_sld_block") and the solvents hexane and/or air ("A_sld_solvent").

The intensity contribution of the hexane $\mathrm{I}_{\mathrm{hexane}}$ was composed of a position ("B_peak_pos"), a scale ("B_scale"), and a half-width at half-maximum (HWHM) of the peak ("B_peak_hwhm"). Likewise, the two silica contributions I_{silica1} and I_{silica2} consisted of a position ("C_peak_pos", "D_peak_pos"), a scale ("C_scale", "D_scale"), and a HWHM ("C_peak_hwhm", "D_peak_hwhm").

Note S8. Assumptions made in the modelling of SasView parameters

i) While a certain amount of drying was expected when transferring the samples from the storage container to the *in-situ* measurement cell, for simplification it was assumed that all samples and their individual positions started with the maximum hexane content.

ii) While the hexane was drying from the silica backbone, the volume shrinkage and hexane evaporation was proportional. This was assumed from photographs taken by the digital microscope camera, showing a transparent sample until maximum shrinkage. Additionally, it was reported in the literature that the initial drying period is evincing an identical loss of volume of the sample and volume of the solvent.[10]

iii) Likewise, the photographs showed a fully shrunken sample which turned translucent. This was an indication of a sample which was not completely filled by hexane, turning into a three-phase system of silica-hexane-air.

iv) Similarly to assumption i), it was assumed that after roughly 12 hours of measurements, the hexane content was negligible.

v) Contrary to a sample which re-expands almost completely after drying, irreversible shrinkage might lead to a change in skeletal density and, thus, in SLD of the silica backbone. Nonetheless, these changes were assumed to be small and negligible.

vi) For simplification, the position of all peaks in the WAXS region did not change, since this region was a convolution of all peaks.

vii) Since the hexane peak was overshadowing the silica peaks, it was not feasible to determine the latter throughout the experiment. Rather the peaks were linked to the scale of the fractal contribution, which was proportional to the volume fraction. This assumed that with a rising volume fraction the sample would get denser and therefore the intensity of these peaks should increase.

viii) With the shrinkage of the material, it was assumed that the size of objects would not be of importance. Therefore, a big and a small cluster would decrease in size equally.

ix) The evaporation of hexane which is the main driving factor of the shrinkage was constant, therefore the changes from one measurement to the next was also constant.

Note S9. Restrictions of the parameters in the SasView model

- "scale": Should be kept constant since this is a scaling factor for the whole model.
- "background": Will be fitted to the lowest intensity values of the measurement.
- "A_scale": Should be fitted if the volume fraction is constant since they depend on each other.
- "A_volfraction": Is a value between 0 and 1 (100 %). The last measurement of a sample was taken from the porosity evaluations of previous studies.[6]
- "A_radius": The radius and its minimum and maximum values were reported previously in literature.[11]

- "A_fractal_dim": The fractal dimension was reported previously in literature.[11] Furthermore, our previous studies showed its restrictions.[1]
- "A_cor_length": The size of clusters and its limitations was reported previously in literature.[11]
- "A_sld_block": The SLD of silica was calculated by SasViews SLD Calculator, using the skeletal density measured in a previous study,[6] and a beam energy of 15 keV.
- "A_sld_solvent": The SLD of the solvent was either that of pure hexane, air, or something in-between.
- "B_scale": With the assumption of a completely dried sample at the end of the measurements, the scale must be 0 for the last measurement and at maximum for the first measurement.
- "B_peak_pos": The position of the hexane peak was taken from a measurement of pure hexane in a glass capillary.
- "B_peak_hwhm": The HWHM was also taken from a measurement of pure hexane.
- "C_scale": It was assumed, that the silica peaks did not change during the evaporation of hexane, but the hexane was overshadowing the contribution of silica. The last measurement without hexane was used to determine these contributions.
- "C_peak_pos": Similarly, the position was fitted once on the last measurement and kept constant throughout the evaluation.
- "C_peak_hwhm": See explanation of "C_scale" and "C_peak_pos".
- "D_scale": See explanation of "C_scale" and "C_peak_pos".
- "D_peak_pos": See explanation of "C_scale" and "C_peak_pos".
- "D_peak_hwhm": See explanation of "C_scale" and "C_peak_pos".

References

1 F. Zemke, E. Scoppola, U. Simon, M. F. Bekheet, W. Wagermaier and A. Gurlo, Springback effect and structural features during the drying of silica aerogels tracked by in-situ synchrotron X-ray scattering, *Sci Rep*, 2022, **12**. DOI: 10.1038/s41598-022-11127-6.

2 G. Benecke, W. Wagermaier, C. Li, M. Schwartzkopf, G. Flucke, R. Hoerth, I. Zizak, M. Burghammer, E. Metwalli, P. Müller-Buschbaum, M. Trebbin, S. Förster, O. Paris, S. V. Roth and P. Fratzl, A customizable software for fast reduction and analysis of large X-ray scattering data sets: applications of the new DPDAK package to small-angle X-ray scattering and grazing-incidence small-angle X-ray scattering, *Journal of applied crystallography*, 2014, **47**, 1797–1803.

3 P. Virtanen, R. Gommers, T. E. Oliphant, M. Haberland, T. Reddy, D. Cournapeau, E. Burovski, P. Peterson, W. Weckesser, J. Bright, S. J. van der Walt, M. Brett, J. Wilson, K. J. Millman, N. Mayorov, A. R. J. Nelson, E. Jones, R. Kern, E. Larson, C. J. Carey, İ. Polat, Y. Feng, E. W. Moore, J. VanderPlas, D. Laxalde, J. Perktold, R. Cimrman, I. Henriksen, E. A. Quintero, C. R. Harris, A. M. Archibald, A. H. Ribeiro, F. Pedregosa and P. van Mulbregt, SciPy 1.0: fundamental algorithms for scientific computing in Python, *Nature methods*, 2020, **17**, 261–272.

4 C. J. Gommes, S. Blacher, B. Goderis and J.-P. Pirard, Phase separation during silica gel formation followed by time-resolved SAXS, *Nuclear Instruments and Methods in Physics Research Section B: Beam Interactions with Materials and Atoms*, 2005, **238**, 141–145.

5 S. H. Madani, I. H. Arellano, J. P. Mata and P. Pendleton, Particle and cluster analyses of silica powders via small angle neutron scattering, *Powder Technology*, 2018, **327**, 96–108.

6 F. Zemke, J. Gonthier, E. Scoppola, U. Simon, M. F. Bekheet, W. Wagermaier and A. Gurlo, Origin of the Springback Effect in Ambient-Pressure-Dried Silica Aerogels: The Effect of Surface Silylation, *Gels (Basel, Switzerland)*, 2023, **9**. DOI: 10.3390/gels9020160.

7 A. Bisson, A. Rigacci, D. Lecomte, E. Rodier and P. Achard, Drying of Silica Gels to Obtain Aerogels:Phenomenology and Basic Techniques, *Drying Technology*, 2003, **21**, 593–628.

8 G. Hura, J. M. Sorenson, R. M. Glaeser and T. Head-Gordon, A high-quality x-ray scattering experiment on liquid water at ambient conditions, *The Journal of chemical physics*, 2000, **113**, 9140–9148.

9 S. J. Rinehart, B. N. Nguyen, R. P. Viggiano, M. A. B. Meador and M. D. Dadmun, Quantitative Evaluation of the Hierarchical Porosity in Polyimide Aerogels and Corresponding Solvated Gels, *ACS applied materials & interfaces*, 2020, **12**, 30457–30465.

10 C. J. Brinker and G. W. Scherer, *Sol-gel science. The physics and chemistry of sol-gel processing*, Academic Press, Inc. An Imprint of Elsevier, Boston [i pozostałe], 1990.

11 T. Woignier, J. Primera, A. Alaoui, P. Dieudonne, L. Duffours, I. Beurroies, S. Calas-Etienne, F. Despestis, A. Faivre and P. Etienne, Fractal Structure in Silica and Composites Aerogels, *Gels (Basel, Switzerland)*, 2020, **7**. DOI: 10.3390/gels7010001.

 This journal is © The Royal Society of Chemistry 2023
